# Model of parafoveal chromatic and luminance temporal contrast sensitivity of humans and monkeys

**DOI:** 10.1167/18.12.1

**Published:** 2018-11-01

**Authors:** Emily C. Gelfand, Gregory D. Horwitz

**Affiliations:** ghorwitz@u.washington.edu; Department of Physiology & Biophysics, Washington National Primate Research Center, University of Washington, Seattle, WA, USA; Department of Physiology & Biophysics, Washington National Primate Research Center, University of Washington, Seattle, WA, USA

**Keywords:** monkey, temporal modulation, contrast sensitivity, detection

## Abstract

Rhesus monkeys are a valuable model for studies of primate visual contrast sensitivity. Their visual systems are similar to that of humans, and they can be trained to perform detection tasks at threshold during neurophysiological recording. However, the stimulus dependence of rhesus monkey contrast sensitivity has not been well characterized. Temporal frequency, color, and retinal eccentricity affect the contrast sensitivity of humans in reasonably well-understood ways. To ask whether these factors affect monkey sensitivity similarly, we measured detection thresholds of two monkeys using a two-alternative, forced-choice task and compared them to thresholds of two human subjects who performed the same task. Stimuli were drifting Gabor patterns that varied in temporal frequency (1–60 Hz), L- and M-cone modulation ratio, and retinal eccentricity (2°–14° from the fovea). Thresholds were fit by a model that assumed a pair of linear detection mechanisms: a luminance contrast detector and a red-green contrast detector. Analysis of model fits indicated that the sensitivity of these mechanisms varied across the visual field, but their temporal and spectral tuning did not. Human and monkey temporal contrast sensitivity was similar across the conditions tested, but monkeys were twofold less sensitive to low-frequency, luminance modulations.

## Introduction

A primary goal of neuroscience is to understand how sensory signals are converted into perceptual experiences. This broad phenomenon can be studied fruitfully through flicker sensitivity. Neurons in the early visual system respond to flicker above the critical flicker fusion frequency, implying a loss of high-frequency information between these neurons and those that mediate perception directly (Lee, Pokorny, Smith, Martin, & Valberg, 1990; Kremers, Lee, & Kaiser, 1992; Yeh, Lee, & Kremers, 1995; Engel, Zhang, & Wandell, 1997; Gur & Snodderly, 1997; Krolak-Salmon et al., 2003; Williams, Mechler, Gordon, Shapley, & Hawken, 2004; Vul & MacLeod, 2006; Jiang, Zhou, & He, 2007; Lee, Sun, & Zucchini, 2007; Falconbridge, Ware, & MacLeod, 2010). In addition, some neurons respond to imperceptible *low*-frequency modulations, demonstrating that information loss is not exclusive to high frequencies (Palmer, Cheng, & Seidemann, 2007; Hass & Horwitz, 2013). The loci and stimulus specificity of information loss in the visual system are largely unknown, and identifying them is an important step toward understanding visual awareness (Crick & Koch, 1998; Carmel, Lavie, & Rees, 2006).

With regard to temporal vision specifically, a significant obstacle to localizing information-processing bottlenecks is that existent neurophysiological and psychophysical measurements are difficult to compare. Several factors contribute. First, psychophysical measurements of temporal contrast sensitivity are made at low contrast, by definition, whereas most neurophysiological studies use high-contrast stimuli. Nonlinearities in neuronal contrast-response functions prevent accurate extrapolation of responses from high to low contrasts. Second, temporal contrast sensitivity varies across the visual field (Sharpe, 1974; Koenderink, Bouma, Bueno de Mesquita, & Slappendel, 1978a; Koenderink, Bouma, Bueno de Mesquita, & Slappendel, 1978b; Virsu, Rovamo, Laurinen, & Nasanen, 1982; Wright & Johnston, 1983; Rovamo & Raninen, 1984; Tyler, 1985; Tyler, 1987; Pointer & Hess, 1989; Snowden & Hess, 1992) and with retinal illumination (De Lange Dzn, 1961; Kelly, 1972; Rovamo & Raninen, 1984; Snowden, Hess, & Waugh, 1995). Neurophysiological and psychophysical measurements are rarely matched for these conditions. Finally, most neurophysiological measurements of flicker sensitivity have been made in animal models, and relatively little is known about the temporal contrast sensitivity of these animals (but see De Valois, Morgan, Polson, Mead, & Hull, 1974; Merigan, 1980).

To help bridge the gap between neurophysiological and psychophysical measurements of temporal contrast sensitivity, we made behavioral measurements in rhesus monkeys—the animal most frequently used to model human visual behavior. Specifically, we used a two-alternative, forced-choice (2AFC) task to measure contrast sensitivity of two rhesus monkeys as a function of three factors: temporal frequency, the relative modulation depth of the long wavelength-sensitive (L) cones and the medium wavelength-sensitive (M) cones (i.e., color direction in the LM plane of cone contrast space), and position in the visual field. We varied color because monkeys are highly sensitive to chromatic modulations under some conditions (Stoughton, Lafer-Sousa, Gagin, & Conway, 2012; Gagin et al., 2014; Lindbloom-Brown, Tait, & Horwitz, 2014). We also varied visual field location because chromatic sensitivity drops steeply with retinal eccentricity in humans (Anderson, Mullen, & Hess, 1991; Mullen, 1991; Stromeyer, Lee, & Eskew, 1992; Mullen & Kingdom, 2002), and most neurophysiological studies probe neurons with parafoveal receptive fields. For comparison, we also measured the temporal contrast sensitivity of two human observers under the same conditions as the monkeys.

To analyze the data, we built a model that described contrast sensitivity across the range of stimulus variations tested. The model was based on three established models, each of which described contrast sensitivity as a function of temporal frequency (Watson, 1986), color direction (Stromeyer, Cole, & Kronauer, 1985), and location in the visual field (Robson & Graham, 1981). These models had not been previously united, but we found that a simple combination predicted thresholds accurately without the need to assume complex interactions among the model parameters.

## Methods

### Subjects

Four subjects participated in this study: the authors (H1, a 23-year-old woman; H2, a 46-year-old man) and 2 nonhuman primates (M1 and M2, both male, *Macaca mulatta*). All procedures used with nonhuman primates were approved by the University of Washington Institutional Animal Care and Use Committee and adhered to the American Physiological Society's Guiding Principles for the Care and Use of Vertebrate Animals in Research and Training. All procedures used with human subjects conformed to the Declaration of Helsinki and the policies of the University of Washington Human Subjects Division. Human subjects provided written, informed consent.

### Displays

All subjects were tested in a room that was dark except for the light from a digital light-processing projector (ProPixx, VPixx Inc., Saint-Bruno, Canada) illuminating a rear projection screen (Da-lite Inc., Warsaw, IN) at 240 Hz. The screen subtended 46° × 26° of visual angle. The center of the screen was 61 cm in front of the subject and matched vertically and horizontally to the subject's eye level. The chromaticity of the display background was (*x* = 0.3, *y* = 0.3), and the luminance was 90 cd/m^2^.

### Psychophysical task

**Figure 1:**
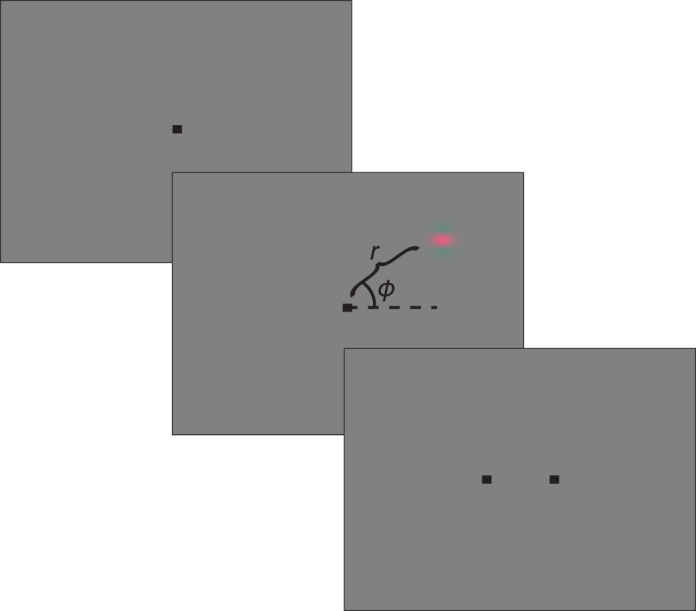
Contrast detection task. Panels from top to bottom show the sequence of events in each trial. Top panel: Subject fixates. Middle panel: Gabor stimulus appears. The horizontal meridian (dotted line), φ (arc), and r (curly bracket) illustrate the polar coordinate system used to describe the location of the stimulus; they were not visible to the subject. Bottom panel: Choice targets appear.

Contrast detection thresholds were measured using a spatial 2AFC contrast detection task. Each trial began with the presentation of a 0.2° × 0.2° black fixation point at the center of the screen (Figure 1). Five hundred milliseconds later, a Gabor stimulus appeared in the left or right hemifield. The fixation point disappeared 100 to 600 ms after the end of the stimulus presentation, and simultaneously, two targets appeared on the horizontal meridian. The subject was then required to indicate within 700 ms whether the stimulus had appeared on the left or right by selecting the corresponding target. Correct responses were accompanied by a tone and, for monkeys, a water reward.

### Testing procedures

Monkey subjects were seated in a testing chair, with their heads stabilized by a head posting device. Eye position was tracked with a scleral search coil (Riverbend Instruments, Birmingham, AL). In 86% of the testing sessions, fixation was required to remain within a 1° × 1° window. In the remaining 14% of the testing sessions, the fixation window was enlarged to a maximum of 1.5° × 1.5°. Targets appeared 2° from the fixation point on the horizontal meridian.

Human subjects performed the same psychophysical task as the monkeys. In 42% (133 of 320) of the testing sessions, the subject's reports were expressed via saccades to the same target locations as the monkeys'. In these sessions, head position was stabilized with a chin rest, eye position was tracked (EyeLink 1000 Plus, SR Research Ltd., Ottawa, Canada), and fixation was enforced. In the other 58% of sessions, subjects indicated their responses with a button box, and eye position was not tracked. The chin rest was used in most but not all of these sessions. Sixty percent of the button box sessions were conducted before the eye tracker sessions.

To examine the effect of response method on detection thresholds, we compared thresholds for 10 different combinations of color direction and temporal frequency, on the horizontal meridian, 5° from the fixation point. Threshold measurements were strongly correlated across response methods (*r* = 0.93 and 0.67 for H1 and H2, respectively) and did not differ significantly for either subject (paired *t*-tests: *p* = 0.86 and *p* = 0.11), indicating that the two response methods yielded similar threshold measurements.

### Stimuli

The stimulus was an upward-drifting, horizontally oriented Gabor, with a spatial frequency of 1 cycle/° and a standard deviation of 0.15°. Stimulus contrast ramped up over 167 ms, remained constant for 334 ms, and then ramped down over 167 ms. The length of the stimulus duration mitigates the effect of the contrast envelope on the temporal frequency power spectrum.

Contrast detection thresholds were measured as a function of three variables: temporal frequency, color direction in the LM plane, and location in the visual field. Temporal frequency and color direction varied within blocks of trials and, on each trial, were selected from a set of two to four combinations that were chosen at the beginning of the block. Stimulus locations in the visual field were fixed within each block. Practice trials at the beginning of each block familiarized the subjects with the stimulus locations. Nevertheless, increases in spatial uncertainty with retinal eccentricity presumably manifest as increases in contrast detection thresholds (Pelli, 1985; Levi, Klein, & Yap, 1987).

Colorimetric calculations were based on the Stockman, MacLeod, and Johnson (1993) 10° cone fundamentals. S-cones were not modulated, and all stimuli were presented at ≥2° from the fovea to avoid peak macular pigment density. L- and M-cone contrasts were defined as

\newcommand{\bialpha}{\boldsymbol{\alpha}}\newcommand{\bibeta}{\boldsymbol{\beta}}\newcommand{\bigamma}{\boldsymbol{\gamma}}\newcommand{\bidelta}{\boldsymbol{\delta}}\newcommand{\bivarepsilon}{\boldsymbol{\varepsilon}}\newcommand{\bizeta}{\boldsymbol{\zeta}}\newcommand{\bieta}{\boldsymbol{\eta}}\newcommand{\bitheta}{\boldsymbol{\theta}}\newcommand{\biiota}{\boldsymbol{\iota}}\newcommand{\bikappa}{\boldsymbol{\kappa}}\newcommand{\bilambda}{\boldsymbol{\lambda}}\newcommand{\bimu}{\boldsymbol{\mu}}\newcommand{\binu}{\boldsymbol{\nu}}\newcommand{\bixi}{\boldsymbol{\xi}}\newcommand{\biomicron}{\boldsymbol{\micron}}\newcommand{\bipi}{\boldsymbol{\pi}}\newcommand{\birho}{\boldsymbol{\rho}}\newcommand{\bisigma}{\boldsymbol{\sigma}}\newcommand{\bitau}{\boldsymbol{\tau}}\newcommand{\biupsilon}{\boldsymbol{\upsilon}}\newcommand{\biphi}{\boldsymbol{\phi}}\newcommand{\bichi}{\boldsymbol{\chi}}\newcommand{\bipsi}{\boldsymbol{\psi}}\newcommand{\biomega}{\boldsymbol{\omega}}\begin{equation}\tag{1}L {\mbox{-}} cone\;contrast = {{{L_{STIM}} - {L_{BACKGROUND}}} \over {{L_{BACKGROUND}}}},\end{equation}

\newcommand{\bialpha}{\boldsymbol{\alpha}}\newcommand{\bibeta}{\boldsymbol{\beta}}\newcommand{\bigamma}{\boldsymbol{\gamma}}\newcommand{\bidelta}{\boldsymbol{\delta}}\newcommand{\bivarepsilon}{\boldsymbol{\varepsilon}}\newcommand{\bizeta}{\boldsymbol{\zeta}}\newcommand{\bieta}{\boldsymbol{\eta}}\newcommand{\bitheta}{\boldsymbol{\theta}}\newcommand{\biiota}{\boldsymbol{\iota}}\newcommand{\bikappa}{\boldsymbol{\kappa}}\newcommand{\bilambda}{\boldsymbol{\lambda}}\newcommand{\bimu}{\boldsymbol{\mu}}\newcommand{\binu}{\boldsymbol{\nu}}\newcommand{\bixi}{\boldsymbol{\xi}}\newcommand{\biomicron}{\boldsymbol{\micron}}\newcommand{\bipi}{\boldsymbol{\pi}}\newcommand{\birho}{\boldsymbol{\rho}}\newcommand{\bisigma}{\boldsymbol{\sigma}}\newcommand{\bitau}{\boldsymbol{\tau}}\newcommand{\biupsilon}{\boldsymbol{\upsilon}}\newcommand{\biphi}{\boldsymbol{\phi}}\newcommand{\bichi}{\boldsymbol{\chi}}\newcommand{\bipsi}{\boldsymbol{\psi}}\newcommand{\biomega}{\boldsymbol{\omega}}\begin{equation}\tag{2}M {\mbox{-}} cone\;contrast = {{{M_{STIM}} - {M_{BACKGROUND}}} \over {{M_{BACKGROUND}}}},\end{equation}

where *L_STIM_* represents the L-cone excitation produced by the peak of the Gabor stimulus and *L_BACKGROUND_* represents the L-cone excitation produced by the background. The quantities *M_STIM_* and *M_BACKGROUND_* are identical except for the M-cones.

Color direction was defined as

\newcommand{\bialpha}{\boldsymbol{\alpha}}\newcommand{\bibeta}{\boldsymbol{\beta}}\newcommand{\bigamma}{\boldsymbol{\gamma}}\newcommand{\bidelta}{\boldsymbol{\delta}}\newcommand{\bivarepsilon}{\boldsymbol{\varepsilon}}\newcommand{\bizeta}{\boldsymbol{\zeta}}\newcommand{\bieta}{\boldsymbol{\eta}}\newcommand{\bitheta}{\boldsymbol{\theta}}\newcommand{\biiota}{\boldsymbol{\iota}}\newcommand{\bikappa}{\boldsymbol{\kappa}}\newcommand{\bilambda}{\boldsymbol{\lambda}}\newcommand{\bimu}{\boldsymbol{\mu}}\newcommand{\binu}{\boldsymbol{\nu}}\newcommand{\bixi}{\boldsymbol{\xi}}\newcommand{\biomicron}{\boldsymbol{\micron}}\newcommand{\bipi}{\boldsymbol{\pi}}\newcommand{\birho}{\boldsymbol{\rho}}\newcommand{\bisigma}{\boldsymbol{\sigma}}\newcommand{\bitau}{\boldsymbol{\tau}}\newcommand{\biupsilon}{\boldsymbol{\upsilon}}\newcommand{\biphi}{\boldsymbol{\phi}}\newcommand{\bichi}{\boldsymbol{\chi}}\newcommand{\bipsi}{\boldsymbol{\psi}}\newcommand{\biomega}{\boldsymbol{\omega}}\begin{equation}\tag{3}{\tan ^{ - 1}}\left( {{{L {\mbox{-}} cone\;contrast} \over {M {\mbox{-}} cone\;contrast}}} \right),\end{equation}

and the modulation amplitude of the stimulus was defined as

\newcommand{\bialpha}{\boldsymbol{\alpha}}\newcommand{\bibeta}{\boldsymbol{\beta}}\newcommand{\bigamma}{\boldsymbol{\gamma}}\newcommand{\bidelta}{\boldsymbol{\delta}}\newcommand{\bivarepsilon}{\boldsymbol{\varepsilon}}\newcommand{\bizeta}{\boldsymbol{\zeta}}\newcommand{\bieta}{\boldsymbol{\eta}}\newcommand{\bitheta}{\boldsymbol{\theta}}\newcommand{\biiota}{\boldsymbol{\iota}}\newcommand{\bikappa}{\boldsymbol{\kappa}}\newcommand{\bilambda}{\boldsymbol{\lambda}}\newcommand{\bimu}{\boldsymbol{\mu}}\newcommand{\binu}{\boldsymbol{\nu}}\newcommand{\bixi}{\boldsymbol{\xi}}\newcommand{\biomicron}{\boldsymbol{\micron}}\newcommand{\bipi}{\boldsymbol{\pi}}\newcommand{\birho}{\boldsymbol{\rho}}\newcommand{\bisigma}{\boldsymbol{\sigma}}\newcommand{\bitau}{\boldsymbol{\tau}}\newcommand{\biupsilon}{\boldsymbol{\upsilon}}\newcommand{\biphi}{\boldsymbol{\phi}}\newcommand{\bichi}{\boldsymbol{\chi}}\newcommand{\bipsi}{\boldsymbol{\psi}}\newcommand{\biomega}{\boldsymbol{\omega}}\begin{equation}\tag{4}\sqrt {L{\mbox{-}}cone\;contras{t^2} + M{\mbox{-}}cone\;contras{t^2}} .\end{equation}

**Figure 2:**
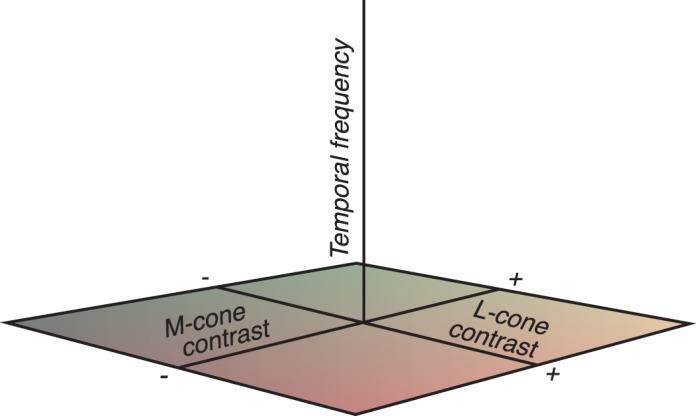
Stimulus space. Each Gabor stimulus is represented by a pair of points that are symmetric with respect to the temporal frequency axis. Points far from this axis have high contrast, and points on the axis have zero contrast.

The color direction and modulation amplitude of a Gabor pattern that modulates the L- and M-cones can be represented as the direction and length, respectively, of a vector in the LM plane of cone contrast space. Temporal frequency can be varied independently of L- and M-cone contrasts and is therefore represented as an orthogonal stimulus dimension. Thus, each Gabor stimulus is represented in a three-dimensional space (Figure 2).

Contrast detection thresholds for each color direction–temporal frequency combination were measured by the QUEST procedure (Watson & Pelli, 1983). The mode of the QUEST function after 40 trials was taken as an estimate of the threshold. The number of threshold measurements from each subject is provided in Table 1.

**Table 1:** Number of threshold measurements per subject. Notes: Color direction and temporal frequency conditions were distributed nearly continuously in the experiment but are binned coarsely in the table. Nonopponent and opponent stimuli are those in which L- and M-cone modulations had the same or opposite sign, respectively.

Subject	Total No. of threshold measurements	Opponent ≤5 Hz	Nonopponent ≤5 Hz	Opponent >5 Hz	Nonopponent >5 Hz
M1	344	56	86	69	133
M2	724	117	193	151	263
H1	220	35	60	39	86
H2	272	41	77	46	108

Within each block of trials, the Gabor stimulus appeared at one of two locations that were mirror symmetric about the vertical meridian. We refer to these location pairs as the location (singular) of the stimulus, because knowing one member of the pair identifies the other. At each location tested, thresholds were first measured with four stimuli: 1 Hz L+M, 1 Hz L−M, 60 Hz L+M, and 60 Hz L−M.

Subsequent color direction–temporal frequency combinations were selected using an adaptive procedure based on Gaussian process regression (Rasmussen, 2004). Before each session, the subject's thresholds were fitted with a nonparametric function that provided threshold predictions for every color direction–temporal frequency combination, along with error estimates associated with these predictions. Color direction–temporal frequency combinations were sampled where the estimated prediction error was greatest. The covariance of the Gaussian process was the product of a Matérn function of log temporal frequency and a periodic function of color direction (MacKay, 1998). Hyperparameters of the covariance function, which specify the variance and length scale of the fitted function, were refit after each block by maximum likelihood. Color direction–temporal frequency combinations for which the predicted threshold was outside of the gamut of the display were not tested.

### Modeling contrast sensitivity: Effects of temporal frequency and color direction

Our model of temporal contrast sensitivity is based on one developed by Watson (1986). The Watson model assumes that detection is mediated by a single, linear bandpass filter that can be described as the difference of two low-pass filters, each with transfer function

\newcommand{\bialpha}{\boldsymbol{\alpha}}\newcommand{\bibeta}{\boldsymbol{\beta}}\newcommand{\bigamma}{\boldsymbol{\gamma}}\newcommand{\bidelta}{\boldsymbol{\delta}}\newcommand{\bivarepsilon}{\boldsymbol{\varepsilon}}\newcommand{\bizeta}{\boldsymbol{\zeta}}\newcommand{\bieta}{\boldsymbol{\eta}}\newcommand{\bitheta}{\boldsymbol{\theta}}\newcommand{\biiota}{\boldsymbol{\iota}}\newcommand{\bikappa}{\boldsymbol{\kappa}}\newcommand{\bilambda}{\boldsymbol{\lambda}}\newcommand{\bimu}{\boldsymbol{\mu}}\newcommand{\binu}{\boldsymbol{\nu}}\newcommand{\bixi}{\boldsymbol{\xi}}\newcommand{\biomicron}{\boldsymbol{\micron}}\newcommand{\bipi}{\boldsymbol{\pi}}\newcommand{\birho}{\boldsymbol{\rho}}\newcommand{\bisigma}{\boldsymbol{\sigma}}\newcommand{\bitau}{\boldsymbol{\tau}}\newcommand{\biupsilon}{\boldsymbol{\upsilon}}\newcommand{\biphi}{\boldsymbol{\phi}}\newcommand{\bichi}{\boldsymbol{\chi}}\newcommand{\bipsi}{\boldsymbol{\psi}}\newcommand{\biomega}{\boldsymbol{\omega}}\begin{equation}\tag{5}{H_1}\left( \omega \right) = {\left( {i2\pi \tau \omega + 1} \right)^{ - n}},\end{equation}

where $\newcommand{\bialpha}{\boldsymbol{\alpha}}\newcommand{\bibeta}{\boldsymbol{\beta}}\newcommand{\bigamma}{\boldsymbol{\gamma}}\newcommand{\bidelta}{\boldsymbol{\delta}}\newcommand{\bivarepsilon}{\boldsymbol{\varepsilon}}\newcommand{\bizeta}{\boldsymbol{\zeta}}\newcommand{\bieta}{\boldsymbol{\eta}}\newcommand{\bitheta}{\boldsymbol{\theta}}\newcommand{\biiota}{\boldsymbol{\iota}}\newcommand{\bikappa}{\boldsymbol{\kappa}}\newcommand{\bilambda}{\boldsymbol{\lambda}}\newcommand{\bimu}{\boldsymbol{\mu}}\newcommand{\binu}{\boldsymbol{\nu}}\newcommand{\bixi}{\boldsymbol{\xi}}\newcommand{\biomicron}{\boldsymbol{\micron}}\newcommand{\bipi}{\boldsymbol{\pi}}\newcommand{\birho}{\boldsymbol{\rho}}\newcommand{\bisigma}{\boldsymbol{\sigma}}\newcommand{\bitau}{\boldsymbol{\tau}}\newcommand{\biupsilon}{\boldsymbol{\upsilon}}\newcommand{\biphi}{\boldsymbol{\phi}}\newcommand{\bichi}{\boldsymbol{\chi}}\newcommand{\bipsi}{\boldsymbol{\psi}}\newcommand{\biomega}{\boldsymbol{\omega}}\tau$ is a time constant, $\newcommand{\bialpha}{\boldsymbol{\alpha}}\newcommand{\bibeta}{\boldsymbol{\beta}}\newcommand{\bigamma}{\boldsymbol{\gamma}}\newcommand{\bidelta}{\boldsymbol{\delta}}\newcommand{\bivarepsilon}{\boldsymbol{\varepsilon}}\newcommand{\bizeta}{\boldsymbol{\zeta}}\newcommand{\bieta}{\boldsymbol{\eta}}\newcommand{\bitheta}{\boldsymbol{\theta}}\newcommand{\biiota}{\boldsymbol{\iota}}\newcommand{\bikappa}{\boldsymbol{\kappa}}\newcommand{\bilambda}{\boldsymbol{\lambda}}\newcommand{\bimu}{\boldsymbol{\mu}}\newcommand{\binu}{\boldsymbol{\nu}}\newcommand{\bixi}{\boldsymbol{\xi}}\newcommand{\biomicron}{\boldsymbol{\micron}}\newcommand{\bipi}{\boldsymbol{\pi}}\newcommand{\birho}{\boldsymbol{\rho}}\newcommand{\bisigma}{\boldsymbol{\sigma}}\newcommand{\bitau}{\boldsymbol{\tau}}\newcommand{\biupsilon}{\boldsymbol{\upsilon}}\newcommand{\biphi}{\boldsymbol{\phi}}\newcommand{\bichi}{\boldsymbol{\chi}}\newcommand{\bipsi}{\boldsymbol{\psi}}\newcommand{\biomega}{\boldsymbol{\omega}}\omega$ is temporal frequency in Hz, and $\newcommand{\bialpha}{\boldsymbol{\alpha}}\newcommand{\bibeta}{\boldsymbol{\beta}}\newcommand{\bigamma}{\boldsymbol{\gamma}}\newcommand{\bidelta}{\boldsymbol{\delta}}\newcommand{\bivarepsilon}{\boldsymbol{\varepsilon}}\newcommand{\bizeta}{\boldsymbol{\zeta}}\newcommand{\bieta}{\boldsymbol{\eta}}\newcommand{\bitheta}{\boldsymbol{\theta}}\newcommand{\biiota}{\boldsymbol{\iota}}\newcommand{\bikappa}{\boldsymbol{\kappa}}\newcommand{\bilambda}{\boldsymbol{\lambda}}\newcommand{\bimu}{\boldsymbol{\mu}}\newcommand{\binu}{\boldsymbol{\nu}}\newcommand{\bixi}{\boldsymbol{\xi}}\newcommand{\biomicron}{\boldsymbol{\micron}}\newcommand{\bipi}{\boldsymbol{\pi}}\newcommand{\birho}{\boldsymbol{\rho}}\newcommand{\bisigma}{\boldsymbol{\sigma}}\newcommand{\bitau}{\boldsymbol{\tau}}\newcommand{\biupsilon}{\boldsymbol{\upsilon}}\newcommand{\biphi}{\boldsymbol{\phi}}\newcommand{\bichi}{\boldsymbol{\chi}}\newcommand{\bipsi}{\boldsymbol{\psi}}\newcommand{\biomega}{\boldsymbol{\omega}}n$ is the number of low-pass stages. The transfer function of the bandpass filter is the difference between the transfer functions of two low-pass filters:

\newcommand{\bialpha}{\boldsymbol{\alpha}}\newcommand{\bibeta}{\boldsymbol{\beta}}\newcommand{\bigamma}{\boldsymbol{\gamma}}\newcommand{\bidelta}{\boldsymbol{\delta}}\newcommand{\bivarepsilon}{\boldsymbol{\varepsilon}}\newcommand{\bizeta}{\boldsymbol{\zeta}}\newcommand{\bieta}{\boldsymbol{\eta}}\newcommand{\bitheta}{\boldsymbol{\theta}}\newcommand{\biiota}{\boldsymbol{\iota}}\newcommand{\bikappa}{\boldsymbol{\kappa}}\newcommand{\bilambda}{\boldsymbol{\lambda}}\newcommand{\bimu}{\boldsymbol{\mu}}\newcommand{\binu}{\boldsymbol{\nu}}\newcommand{\bixi}{\boldsymbol{\xi}}\newcommand{\biomicron}{\boldsymbol{\micron}}\newcommand{\bipi}{\boldsymbol{\pi}}\newcommand{\birho}{\boldsymbol{\rho}}\newcommand{\bisigma}{\boldsymbol{\sigma}}\newcommand{\bitau}{\boldsymbol{\tau}}\newcommand{\biupsilon}{\boldsymbol{\upsilon}}\newcommand{\biphi}{\boldsymbol{\phi}}\newcommand{\bichi}{\boldsymbol{\chi}}\newcommand{\bipsi}{\boldsymbol{\psi}}\newcommand{\biomega}{\boldsymbol{\omega}}\begin{equation}\tag{6}H\left( \omega \right) = \xi \left( {{H_1}\left( \omega \right) - \zeta {H_2}\left( \omega \right)} \right),\end{equation}

where $\newcommand{\bialpha}{\boldsymbol{\alpha}}\newcommand{\bibeta}{\boldsymbol{\beta}}\newcommand{\bigamma}{\boldsymbol{\gamma}}\newcommand{\bidelta}{\boldsymbol{\delta}}\newcommand{\bivarepsilon}{\boldsymbol{\varepsilon}}\newcommand{\bizeta}{\boldsymbol{\zeta}}\newcommand{\bieta}{\boldsymbol{\eta}}\newcommand{\bitheta}{\boldsymbol{\theta}}\newcommand{\biiota}{\boldsymbol{\iota}}\newcommand{\bikappa}{\boldsymbol{\kappa}}\newcommand{\bilambda}{\boldsymbol{\lambda}}\newcommand{\bimu}{\boldsymbol{\mu}}\newcommand{\binu}{\boldsymbol{\nu}}\newcommand{\bixi}{\boldsymbol{\xi}}\newcommand{\biomicron}{\boldsymbol{\micron}}\newcommand{\bipi}{\boldsymbol{\pi}}\newcommand{\birho}{\boldsymbol{\rho}}\newcommand{\bisigma}{\boldsymbol{\sigma}}\newcommand{\bitau}{\boldsymbol{\tau}}\newcommand{\biupsilon}{\boldsymbol{\upsilon}}\newcommand{\biphi}{\boldsymbol{\phi}}\newcommand{\bichi}{\boldsymbol{\chi}}\newcommand{\bipsi}{\boldsymbol{\psi}}\newcommand{\biomega}{\boldsymbol{\omega}}{H_1}\left( \omega \right)$ and $\newcommand{\bialpha}{\boldsymbol{\alpha}}\newcommand{\bibeta}{\boldsymbol{\beta}}\newcommand{\bigamma}{\boldsymbol{\gamma}}\newcommand{\bidelta}{\boldsymbol{\delta}}\newcommand{\bivarepsilon}{\boldsymbol{\varepsilon}}\newcommand{\bizeta}{\boldsymbol{\zeta}}\newcommand{\bieta}{\boldsymbol{\eta}}\newcommand{\bitheta}{\boldsymbol{\theta}}\newcommand{\biiota}{\boldsymbol{\iota}}\newcommand{\bikappa}{\boldsymbol{\kappa}}\newcommand{\bilambda}{\boldsymbol{\lambda}}\newcommand{\bimu}{\boldsymbol{\mu}}\newcommand{\binu}{\boldsymbol{\nu}}\newcommand{\bixi}{\boldsymbol{\xi}}\newcommand{\biomicron}{\boldsymbol{\micron}}\newcommand{\bipi}{\boldsymbol{\pi}}\newcommand{\birho}{\boldsymbol{\rho}}\newcommand{\bisigma}{\boldsymbol{\sigma}}\newcommand{\bitau}{\boldsymbol{\tau}}\newcommand{\biupsilon}{\boldsymbol{\upsilon}}\newcommand{\biphi}{\boldsymbol{\phi}}\newcommand{\bichi}{\boldsymbol{\chi}}\newcommand{\bipsi}{\boldsymbol{\psi}}\newcommand{\biomega}{\boldsymbol{\omega}}{H_2}\left( \omega \right)$ are the transfer functions of the low-pass filters defined by Equation 5, $\newcommand{\bialpha}{\boldsymbol{\alpha}}\newcommand{\bibeta}{\boldsymbol{\beta}}\newcommand{\bigamma}{\boldsymbol{\gamma}}\newcommand{\bidelta}{\boldsymbol{\delta}}\newcommand{\bivarepsilon}{\boldsymbol{\varepsilon}}\newcommand{\bizeta}{\boldsymbol{\zeta}}\newcommand{\bieta}{\boldsymbol{\eta}}\newcommand{\bitheta}{\boldsymbol{\theta}}\newcommand{\biiota}{\boldsymbol{\iota}}\newcommand{\bikappa}{\boldsymbol{\kappa}}\newcommand{\bilambda}{\boldsymbol{\lambda}}\newcommand{\bimu}{\boldsymbol{\mu}}\newcommand{\binu}{\boldsymbol{\nu}}\newcommand{\bixi}{\boldsymbol{\xi}}\newcommand{\biomicron}{\boldsymbol{\micron}}\newcommand{\bipi}{\boldsymbol{\pi}}\newcommand{\birho}{\boldsymbol{\rho}}\newcommand{\bisigma}{\boldsymbol{\sigma}}\newcommand{\bitau}{\boldsymbol{\tau}}\newcommand{\biupsilon}{\boldsymbol{\upsilon}}\newcommand{\biphi}{\boldsymbol{\phi}}\newcommand{\bichi}{\boldsymbol{\chi}}\newcommand{\bipsi}{\boldsymbol{\psi}}\newcommand{\biomega}{\boldsymbol{\omega}}\xi$ is a gain parameter, and $\newcommand{\bialpha}{\boldsymbol{\alpha}}\newcommand{\bibeta}{\boldsymbol{\beta}}\newcommand{\bigamma}{\boldsymbol{\gamma}}\newcommand{\bidelta}{\boldsymbol{\delta}}\newcommand{\bivarepsilon}{\boldsymbol{\varepsilon}}\newcommand{\bizeta}{\boldsymbol{\zeta}}\newcommand{\bieta}{\boldsymbol{\eta}}\newcommand{\bitheta}{\boldsymbol{\theta}}\newcommand{\biiota}{\boldsymbol{\iota}}\newcommand{\bikappa}{\boldsymbol{\kappa}}\newcommand{\bilambda}{\boldsymbol{\lambda}}\newcommand{\bimu}{\boldsymbol{\mu}}\newcommand{\binu}{\boldsymbol{\nu}}\newcommand{\bixi}{\boldsymbol{\xi}}\newcommand{\biomicron}{\boldsymbol{\micron}}\newcommand{\bipi}{\boldsymbol{\pi}}\newcommand{\birho}{\boldsymbol{\rho}}\newcommand{\bisigma}{\boldsymbol{\sigma}}\newcommand{\bitau}{\boldsymbol{\tau}}\newcommand{\biupsilon}{\boldsymbol{\upsilon}}\newcommand{\biphi}{\boldsymbol{\phi}}\newcommand{\bichi}{\boldsymbol{\chi}}\newcommand{\bipsi}{\boldsymbol{\psi}}\newcommand{\biomega}{\boldsymbol{\omega}}\zeta$ controls the transience of the bandpass filter. When $\newcommand{\bialpha}{\boldsymbol{\alpha}}\newcommand{\bibeta}{\boldsymbol{\beta}}\newcommand{\bigamma}{\boldsymbol{\gamma}}\newcommand{\bidelta}{\boldsymbol{\delta}}\newcommand{\bivarepsilon}{\boldsymbol{\varepsilon}}\newcommand{\bizeta}{\boldsymbol{\zeta}}\newcommand{\bieta}{\boldsymbol{\eta}}\newcommand{\bitheta}{\boldsymbol{\theta}}\newcommand{\biiota}{\boldsymbol{\iota}}\newcommand{\bikappa}{\boldsymbol{\kappa}}\newcommand{\bilambda}{\boldsymbol{\lambda}}\newcommand{\bimu}{\boldsymbol{\mu}}\newcommand{\binu}{\boldsymbol{\nu}}\newcommand{\bixi}{\boldsymbol{\xi}}\newcommand{\biomicron}{\boldsymbol{\micron}}\newcommand{\bipi}{\boldsymbol{\pi}}\newcommand{\birho}{\boldsymbol{\rho}}\newcommand{\bisigma}{\boldsymbol{\sigma}}\newcommand{\bitau}{\boldsymbol{\tau}}\newcommand{\biupsilon}{\boldsymbol{\upsilon}}\newcommand{\biphi}{\boldsymbol{\phi}}\newcommand{\bichi}{\boldsymbol{\chi}}\newcommand{\bipsi}{\boldsymbol{\psi}}\newcommand{\biomega}{\boldsymbol{\omega}}\zeta = 0$, the filter is low pass, and when $\newcommand{\bialpha}{\boldsymbol{\alpha}}\newcommand{\bibeta}{\boldsymbol{\beta}}\newcommand{\bigamma}{\boldsymbol{\gamma}}\newcommand{\bidelta}{\boldsymbol{\delta}}\newcommand{\bivarepsilon}{\boldsymbol{\varepsilon}}\newcommand{\bizeta}{\boldsymbol{\zeta}}\newcommand{\bieta}{\boldsymbol{\eta}}\newcommand{\bitheta}{\boldsymbol{\theta}}\newcommand{\biiota}{\boldsymbol{\iota}}\newcommand{\bikappa}{\boldsymbol{\kappa}}\newcommand{\bilambda}{\boldsymbol{\lambda}}\newcommand{\bimu}{\boldsymbol{\mu}}\newcommand{\binu}{\boldsymbol{\nu}}\newcommand{\bixi}{\boldsymbol{\xi}}\newcommand{\biomicron}{\boldsymbol{\micron}}\newcommand{\bipi}{\boldsymbol{\pi}}\newcommand{\birho}{\boldsymbol{\rho}}\newcommand{\bisigma}{\boldsymbol{\sigma}}\newcommand{\bitau}{\boldsymbol{\tau}}\newcommand{\biupsilon}{\boldsymbol{\upsilon}}\newcommand{\biphi}{\boldsymbol{\phi}}\newcommand{\bichi}{\boldsymbol{\chi}}\newcommand{\bipsi}{\boldsymbol{\psi}}\newcommand{\biomega}{\boldsymbol{\omega}}\zeta \ \gt \ 0$, the filter is bandpass.

We extended this model to describe contrast detection thresholds across color directions in the LM plane. We assumed that detection is mediated by two linear mechanisms whose outputs are squared and summed. As a consequence, detection contours at any temporal frequency were constrained to be elliptical. We did not assume that the luminance and chromatic mechanisms were orthogonal. Therefore, the orientation of detection ellipses in the LM plane could, and in general did, change with temporal frequency.

One of the mechanisms (RG) was assumed to respond to the difference between L- and M-cone contrasts. The second mechanism (LUM) was assumed to respond to a weighted sum of L- and M-cone contrast. The sensitivity of each mechanism at frequency $\newcommand{\bialpha}{\boldsymbol{\alpha}}\newcommand{\bibeta}{\boldsymbol{\beta}}\newcommand{\bigamma}{\boldsymbol{\gamma}}\newcommand{\bidelta}{\boldsymbol{\delta}}\newcommand{\bivarepsilon}{\boldsymbol{\varepsilon}}\newcommand{\bizeta}{\boldsymbol{\zeta}}\newcommand{\bieta}{\boldsymbol{\eta}}\newcommand{\bitheta}{\boldsymbol{\theta}}\newcommand{\biiota}{\boldsymbol{\iota}}\newcommand{\bikappa}{\boldsymbol{\kappa}}\newcommand{\bilambda}{\boldsymbol{\lambda}}\newcommand{\bimu}{\boldsymbol{\mu}}\newcommand{\binu}{\boldsymbol{\nu}}\newcommand{\bixi}{\boldsymbol{\xi}}\newcommand{\biomicron}{\boldsymbol{\micron}}\newcommand{\bipi}{\boldsymbol{\pi}}\newcommand{\birho}{\boldsymbol{\rho}}\newcommand{\bisigma}{\boldsymbol{\sigma}}\newcommand{\bitau}{\boldsymbol{\tau}}\newcommand{\biupsilon}{\boldsymbol{\upsilon}}\newcommand{\biphi}{\boldsymbol{\phi}}\newcommand{\bichi}{\boldsymbol{\chi}}\newcommand{\bipsi}{\boldsymbol{\psi}}\newcommand{\biomega}{\boldsymbol{\omega}}\omega$ was *H_RG_*($\newcommand{\bialpha}{\boldsymbol{\alpha}}\newcommand{\bibeta}{\boldsymbol{\beta}}\newcommand{\bigamma}{\boldsymbol{\gamma}}\newcommand{\bidelta}{\boldsymbol{\delta}}\newcommand{\bivarepsilon}{\boldsymbol{\varepsilon}}\newcommand{\bizeta}{\boldsymbol{\zeta}}\newcommand{\bieta}{\boldsymbol{\eta}}\newcommand{\bitheta}{\boldsymbol{\theta}}\newcommand{\biiota}{\boldsymbol{\iota}}\newcommand{\bikappa}{\boldsymbol{\kappa}}\newcommand{\bilambda}{\boldsymbol{\lambda}}\newcommand{\bimu}{\boldsymbol{\mu}}\newcommand{\binu}{\boldsymbol{\nu}}\newcommand{\bixi}{\boldsymbol{\xi}}\newcommand{\biomicron}{\boldsymbol{\micron}}\newcommand{\bipi}{\boldsymbol{\pi}}\newcommand{\birho}{\boldsymbol{\rho}}\newcommand{\bisigma}{\boldsymbol{\sigma}}\newcommand{\bitau}{\boldsymbol{\tau}}\newcommand{\biupsilon}{\boldsymbol{\upsilon}}\newcommand{\biphi}{\boldsymbol{\phi}}\newcommand{\bichi}{\boldsymbol{\chi}}\newcommand{\bipsi}{\boldsymbol{\psi}}\newcommand{\biomega}{\boldsymbol{\omega}}\omega$) and *H_LUM_*($\newcommand{\bialpha}{\boldsymbol{\alpha}}\newcommand{\bibeta}{\boldsymbol{\beta}}\newcommand{\bigamma}{\boldsymbol{\gamma}}\newcommand{\bidelta}{\boldsymbol{\delta}}\newcommand{\bivarepsilon}{\boldsymbol{\varepsilon}}\newcommand{\bizeta}{\boldsymbol{\zeta}}\newcommand{\bieta}{\boldsymbol{\eta}}\newcommand{\bitheta}{\boldsymbol{\theta}}\newcommand{\biiota}{\boldsymbol{\iota}}\newcommand{\bikappa}{\boldsymbol{\kappa}}\newcommand{\bilambda}{\boldsymbol{\lambda}}\newcommand{\bimu}{\boldsymbol{\mu}}\newcommand{\binu}{\boldsymbol{\nu}}\newcommand{\bixi}{\boldsymbol{\xi}}\newcommand{\biomicron}{\boldsymbol{\micron}}\newcommand{\bipi}{\boldsymbol{\pi}}\newcommand{\birho}{\boldsymbol{\rho}}\newcommand{\bisigma}{\boldsymbol{\sigma}}\newcommand{\bitau}{\boldsymbol{\tau}}\newcommand{\biupsilon}{\boldsymbol{\upsilon}}\newcommand{\biphi}{\boldsymbol{\phi}}\newcommand{\bichi}{\boldsymbol{\chi}}\newcommand{\bipsi}{\boldsymbol{\psi}}\newcommand{\biomega}{\boldsymbol{\omega}}\omega$), which are transfer functions that conform to the Watson (1986) model but have different parameter values. The predicted contrast sensitivity across directions in the LM plane was therefore

\newcommand{\bialpha}{\boldsymbol{\alpha}}\newcommand{\bibeta}{\boldsymbol{\beta}}\newcommand{\bigamma}{\boldsymbol{\gamma}}\newcommand{\bidelta}{\boldsymbol{\delta}}\newcommand{\bivarepsilon}{\boldsymbol{\varepsilon}}\newcommand{\bizeta}{\boldsymbol{\zeta}}\newcommand{\bieta}{\boldsymbol{\eta}}\newcommand{\bitheta}{\boldsymbol{\theta}}\newcommand{\biiota}{\boldsymbol{\iota}}\newcommand{\bikappa}{\boldsymbol{\kappa}}\newcommand{\bilambda}{\boldsymbol{\lambda}}\newcommand{\bimu}{\boldsymbol{\mu}}\newcommand{\binu}{\boldsymbol{\nu}}\newcommand{\bixi}{\boldsymbol{\xi}}\newcommand{\biomicron}{\boldsymbol{\micron}}\newcommand{\bipi}{\boldsymbol{\pi}}\newcommand{\birho}{\boldsymbol{\rho}}\newcommand{\bisigma}{\boldsymbol{\sigma}}\newcommand{\bitau}{\boldsymbol{\tau}}\newcommand{\biupsilon}{\boldsymbol{\upsilon}}\newcommand{\biphi}{\boldsymbol{\phi}}\newcommand{\bichi}{\boldsymbol{\chi}}\newcommand{\bipsi}{\boldsymbol{\psi}}\newcommand{\biomega}{\boldsymbol{\omega}}\begin{equation}\tag{7}Contrast\;sensitivity = \sqrt {{{\left( {{H_{RG}}\left( \omega \right)\left[ {\cos \left( {{{3\pi } \over 4}} \right)L + \sin \left( {{{3\pi } \over 4}} \right)M} \right]} \right)}^2} \atop + {{\left( {{H_{LUM}}\left( \omega \right)\left[ {\cos \left( \theta \right)L + \sin \left( \theta \right)M} \right]} \right)}^2}} ,\end{equation}

where *L* and *M* are cone contrasts normalized so that *L^2^* + *M^2^* = 1, and *θ* is a fitted parameter indicating the relative weighting of L- and M-cones to the LUM mechanism. The RG mechanism was assumed to weight L- and M-cone signals equally (Stromeyer et al., 1985; Gegenfurtner & Hawken, 1995; Stromeyer, Kronauer, Chaparro, & Eskew, 1995; Sankeralli & Mullen, 1996). Contrast threshold was defined as the reciprocal of contrast sensitivity.

### Modeling contrast sensitivity: Effects of stimulus position in the visual field

The preceding model describes contrast sensitivity at individual locations in the visual field. To capture differences in contrast sensitivity across the visual field, we extended the model. Visual field locations were represented in polar coordinates, where *r* is the eccentricity of the stimulus in degrees of visual angle, and *φ* is the position of the stimulus in the plane of the screen, relative to the horizontal meridian (Figure 1). These parameters can be written as

\newcommand{\bialpha}{\boldsymbol{\alpha}}\newcommand{\bibeta}{\boldsymbol{\beta}}\newcommand{\bigamma}{\boldsymbol{\gamma}}\newcommand{\bidelta}{\boldsymbol{\delta}}\newcommand{\bivarepsilon}{\boldsymbol{\varepsilon}}\newcommand{\bizeta}{\boldsymbol{\zeta}}\newcommand{\bieta}{\boldsymbol{\eta}}\newcommand{\bitheta}{\boldsymbol{\theta}}\newcommand{\biiota}{\boldsymbol{\iota}}\newcommand{\bikappa}{\boldsymbol{\kappa}}\newcommand{\bilambda}{\boldsymbol{\lambda}}\newcommand{\bimu}{\boldsymbol{\mu}}\newcommand{\binu}{\boldsymbol{\nu}}\newcommand{\bixi}{\boldsymbol{\xi}}\newcommand{\biomicron}{\boldsymbol{\micron}}\newcommand{\bipi}{\boldsymbol{\pi}}\newcommand{\birho}{\boldsymbol{\rho}}\newcommand{\bisigma}{\boldsymbol{\sigma}}\newcommand{\bitau}{\boldsymbol{\tau}}\newcommand{\biupsilon}{\boldsymbol{\upsilon}}\newcommand{\biphi}{\boldsymbol{\phi}}\newcommand{\bichi}{\boldsymbol{\chi}}\newcommand{\bipsi}{\boldsymbol{\psi}}\newcommand{\biomega}{\boldsymbol{\omega}}\begin{equation}\tag{8}r = \sqrt {{h^2} + {v^2}} \end{equation}

\newcommand{\bialpha}{\boldsymbol{\alpha}}\newcommand{\bibeta}{\boldsymbol{\beta}}\newcommand{\bigamma}{\boldsymbol{\gamma}}\newcommand{\bidelta}{\boldsymbol{\delta}}\newcommand{\bivarepsilon}{\boldsymbol{\varepsilon}}\newcommand{\bizeta}{\boldsymbol{\zeta}}\newcommand{\bieta}{\boldsymbol{\eta}}\newcommand{\bitheta}{\boldsymbol{\theta}}\newcommand{\biiota}{\boldsymbol{\iota}}\newcommand{\bikappa}{\boldsymbol{\kappa}}\newcommand{\bilambda}{\boldsymbol{\lambda}}\newcommand{\bimu}{\boldsymbol{\mu}}\newcommand{\binu}{\boldsymbol{\nu}}\newcommand{\bixi}{\boldsymbol{\xi}}\newcommand{\biomicron}{\boldsymbol{\micron}}\newcommand{\bipi}{\boldsymbol{\pi}}\newcommand{\birho}{\boldsymbol{\rho}}\newcommand{\bisigma}{\boldsymbol{\sigma}}\newcommand{\bitau}{\boldsymbol{\tau}}\newcommand{\biupsilon}{\boldsymbol{\upsilon}}\newcommand{\biphi}{\boldsymbol{\phi}}\newcommand{\bichi}{\boldsymbol{\chi}}\newcommand{\bipsi}{\boldsymbol{\psi}}\newcommand{\biomega}{\boldsymbol{\omega}}\begin{equation}\tag{9}\varphi = {\tan ^{ - 1}}{(v) \over (h)},\end{equation}

where *h* and *v* are the horizontal and vertical positions, respectively, of the stimulus in degrees of visual angle relative to the fixation point.

As described in the Results section, we tested several parametric forms of the relationship between $\newcommand{\bialpha}{\boldsymbol{\alpha}}\newcommand{\bibeta}{\boldsymbol{\beta}}\newcommand{\bigamma}{\boldsymbol{\gamma}}\newcommand{\bidelta}{\boldsymbol{\delta}}\newcommand{\bivarepsilon}{\boldsymbol{\varepsilon}}\newcommand{\bizeta}{\boldsymbol{\zeta}}\newcommand{\bieta}{\boldsymbol{\eta}}\newcommand{\bitheta}{\boldsymbol{\theta}}\newcommand{\biiota}{\boldsymbol{\iota}}\newcommand{\bikappa}{\boldsymbol{\kappa}}\newcommand{\bilambda}{\boldsymbol{\lambda}}\newcommand{\bimu}{\boldsymbol{\mu}}\newcommand{\binu}{\boldsymbol{\nu}}\newcommand{\bixi}{\boldsymbol{\xi}}\newcommand{\biomicron}{\boldsymbol{\micron}}\newcommand{\bipi}{\boldsymbol{\pi}}\newcommand{\birho}{\boldsymbol{\rho}}\newcommand{\bisigma}{\boldsymbol{\sigma}}\newcommand{\bitau}{\boldsymbol{\tau}}\newcommand{\biupsilon}{\boldsymbol{\upsilon}}\newcommand{\biphi}{\boldsymbol{\phi}}\newcommand{\bichi}{\boldsymbol{\chi}}\newcommand{\bipsi}{\boldsymbol{\psi}}\newcommand{\biomega}{\boldsymbol{\omega}}{\xi _{LUM}}$ and $\newcommand{\bialpha}{\boldsymbol{\alpha}}\newcommand{\bibeta}{\boldsymbol{\beta}}\newcommand{\bigamma}{\boldsymbol{\gamma}}\newcommand{\bidelta}{\boldsymbol{\delta}}\newcommand{\bivarepsilon}{\boldsymbol{\varepsilon}}\newcommand{\bizeta}{\boldsymbol{\zeta}}\newcommand{\bieta}{\boldsymbol{\eta}}\newcommand{\bitheta}{\boldsymbol{\theta}}\newcommand{\biiota}{\boldsymbol{\iota}}\newcommand{\bikappa}{\boldsymbol{\kappa}}\newcommand{\bilambda}{\boldsymbol{\lambda}}\newcommand{\bimu}{\boldsymbol{\mu}}\newcommand{\binu}{\boldsymbol{\nu}}\newcommand{\bixi}{\boldsymbol{\xi}}\newcommand{\biomicron}{\boldsymbol{\micron}}\newcommand{\bipi}{\boldsymbol{\pi}}\newcommand{\birho}{\boldsymbol{\rho}}\newcommand{\bisigma}{\boldsymbol{\sigma}}\newcommand{\bitau}{\boldsymbol{\tau}}\newcommand{\biupsilon}{\boldsymbol{\upsilon}}\newcommand{\biphi}{\boldsymbol{\phi}}\newcommand{\bichi}{\boldsymbol{\chi}}\newcommand{\bipsi}{\boldsymbol{\psi}}\newcommand{\biomega}{\boldsymbol{\omega}}{\xi _{RG}}$ (Equation 6) and (*r*, *φ*). The general form of the dependence was

\newcommand{\bialpha}{\boldsymbol{\alpha}}\newcommand{\bibeta}{\boldsymbol{\beta}}\newcommand{\bigamma}{\boldsymbol{\gamma}}\newcommand{\bidelta}{\boldsymbol{\delta}}\newcommand{\bivarepsilon}{\boldsymbol{\varepsilon}}\newcommand{\bizeta}{\boldsymbol{\zeta}}\newcommand{\bieta}{\boldsymbol{\eta}}\newcommand{\bitheta}{\boldsymbol{\theta}}\newcommand{\biiota}{\boldsymbol{\iota}}\newcommand{\bikappa}{\boldsymbol{\kappa}}\newcommand{\bilambda}{\boldsymbol{\lambda}}\newcommand{\bimu}{\boldsymbol{\mu}}\newcommand{\binu}{\boldsymbol{\nu}}\newcommand{\bixi}{\boldsymbol{\xi}}\newcommand{\biomicron}{\boldsymbol{\micron}}\newcommand{\bipi}{\boldsymbol{\pi}}\newcommand{\birho}{\boldsymbol{\rho}}\newcommand{\bisigma}{\boldsymbol{\sigma}}\newcommand{\bitau}{\boldsymbol{\tau}}\newcommand{\biupsilon}{\boldsymbol{\upsilon}}\newcommand{\biphi}{\boldsymbol{\phi}}\newcommand{\bichi}{\boldsymbol{\chi}}\newcommand{\bipsi}{\boldsymbol{\psi}}\newcommand{\biomega}{\boldsymbol{\omega}}\begin{equation}\tag{10}{\log _{10}}\left( \xi \right) = {b_0} + {b_1}r + {b_2}r\cos \left( {2\varphi } \right) + {b_3}r\sin \left( {2\varphi } \right),\!\end{equation}

where $\newcommand{\bialpha}{\boldsymbol{\alpha}}\newcommand{\bibeta}{\boldsymbol{\beta}}\newcommand{\bigamma}{\boldsymbol{\gamma}}\newcommand{\bidelta}{\boldsymbol{\delta}}\newcommand{\bivarepsilon}{\boldsymbol{\varepsilon}}\newcommand{\bizeta}{\boldsymbol{\zeta}}\newcommand{\bieta}{\boldsymbol{\eta}}\newcommand{\bitheta}{\boldsymbol{\theta}}\newcommand{\biiota}{\boldsymbol{\iota}}\newcommand{\bikappa}{\boldsymbol{\kappa}}\newcommand{\bilambda}{\boldsymbol{\lambda}}\newcommand{\bimu}{\boldsymbol{\mu}}\newcommand{\binu}{\boldsymbol{\nu}}\newcommand{\bixi}{\boldsymbol{\xi}}\newcommand{\biomicron}{\boldsymbol{\micron}}\newcommand{\bipi}{\boldsymbol{\pi}}\newcommand{\birho}{\boldsymbol{\rho}}\newcommand{\bisigma}{\boldsymbol{\sigma}}\newcommand{\bitau}{\boldsymbol{\tau}}\newcommand{\biupsilon}{\boldsymbol{\upsilon}}\newcommand{\biphi}{\boldsymbol{\phi}}\newcommand{\bichi}{\boldsymbol{\chi}}\newcommand{\bipsi}{\boldsymbol{\psi}}\newcommand{\biomega}{\boldsymbol{\omega}}\xi$ represents $\newcommand{\bialpha}{\boldsymbol{\alpha}}\newcommand{\bibeta}{\boldsymbol{\beta}}\newcommand{\bigamma}{\boldsymbol{\gamma}}\newcommand{\bidelta}{\boldsymbol{\delta}}\newcommand{\bivarepsilon}{\boldsymbol{\varepsilon}}\newcommand{\bizeta}{\boldsymbol{\zeta}}\newcommand{\bieta}{\boldsymbol{\eta}}\newcommand{\bitheta}{\boldsymbol{\theta}}\newcommand{\biiota}{\boldsymbol{\iota}}\newcommand{\bikappa}{\boldsymbol{\kappa}}\newcommand{\bilambda}{\boldsymbol{\lambda}}\newcommand{\bimu}{\boldsymbol{\mu}}\newcommand{\binu}{\boldsymbol{\nu}}\newcommand{\bixi}{\boldsymbol{\xi}}\newcommand{\biomicron}{\boldsymbol{\micron}}\newcommand{\bipi}{\boldsymbol{\pi}}\newcommand{\birho}{\boldsymbol{\rho}}\newcommand{\bisigma}{\boldsymbol{\sigma}}\newcommand{\bitau}{\boldsymbol{\tau}}\newcommand{\biupsilon}{\boldsymbol{\upsilon}}\newcommand{\biphi}{\boldsymbol{\phi}}\newcommand{\bichi}{\boldsymbol{\chi}}\newcommand{\bipsi}{\boldsymbol{\psi}}\newcommand{\biomega}{\boldsymbol{\omega}}{\xi _{LUM}}$ or $\newcommand{\bialpha}{\boldsymbol{\alpha}}\newcommand{\bibeta}{\boldsymbol{\beta}}\newcommand{\bigamma}{\boldsymbol{\gamma}}\newcommand{\bidelta}{\boldsymbol{\delta}}\newcommand{\bivarepsilon}{\boldsymbol{\varepsilon}}\newcommand{\bizeta}{\boldsymbol{\zeta}}\newcommand{\bieta}{\boldsymbol{\eta}}\newcommand{\bitheta}{\boldsymbol{\theta}}\newcommand{\biiota}{\boldsymbol{\iota}}\newcommand{\bikappa}{\boldsymbol{\kappa}}\newcommand{\bilambda}{\boldsymbol{\lambda}}\newcommand{\bimu}{\boldsymbol{\mu}}\newcommand{\binu}{\boldsymbol{\nu}}\newcommand{\bixi}{\boldsymbol{\xi}}\newcommand{\biomicron}{\boldsymbol{\micron}}\newcommand{\bipi}{\boldsymbol{\pi}}\newcommand{\birho}{\boldsymbol{\rho}}\newcommand{\bisigma}{\boldsymbol{\sigma}}\newcommand{\bitau}{\boldsymbol{\tau}}\newcommand{\biupsilon}{\boldsymbol{\upsilon}}\newcommand{\biphi}{\boldsymbol{\phi}}\newcommand{\bichi}{\boldsymbol{\chi}}\newcommand{\bipsi}{\boldsymbol{\psi}}\newcommand{\biomega}{\boldsymbol{\omega}}{\xi _{RG}}$, which govern the sensitivity of the LUM and RG mechanisms, respectively. Setting *φ* = 0 shows that $\newcommand{\bialpha}{\boldsymbol{\alpha}}\newcommand{\bibeta}{\boldsymbol{\beta}}\newcommand{\bigamma}{\boldsymbol{\gamma}}\newcommand{\bidelta}{\boldsymbol{\delta}}\newcommand{\bivarepsilon}{\boldsymbol{\varepsilon}}\newcommand{\bizeta}{\boldsymbol{\zeta}}\newcommand{\bieta}{\boldsymbol{\eta}}\newcommand{\bitheta}{\boldsymbol{\theta}}\newcommand{\biiota}{\boldsymbol{\iota}}\newcommand{\bikappa}{\boldsymbol{\kappa}}\newcommand{\bilambda}{\boldsymbol{\lambda}}\newcommand{\bimu}{\boldsymbol{\mu}}\newcommand{\binu}{\boldsymbol{\nu}}\newcommand{\bixi}{\boldsymbol{\xi}}\newcommand{\biomicron}{\boldsymbol{\micron}}\newcommand{\bipi}{\boldsymbol{\pi}}\newcommand{\birho}{\boldsymbol{\rho}}\newcommand{\bisigma}{\boldsymbol{\sigma}}\newcommand{\bitau}{\boldsymbol{\tau}}\newcommand{\biupsilon}{\boldsymbol{\upsilon}}\newcommand{\biphi}{\boldsymbol{\phi}}\newcommand{\bichi}{\boldsymbol{\chi}}\newcommand{\bipsi}{\boldsymbol{\psi}}\newcommand{\biomega}{\boldsymbol{\omega}}\xi$ changes with slope (*b_1_* + *b_2_*) along the horizontal meridian, and setting *φ* = ±π/2 shows that $\newcommand{\bialpha}{\boldsymbol{\alpha}}\newcommand{\bibeta}{\boldsymbol{\beta}}\newcommand{\bigamma}{\boldsymbol{\gamma}}\newcommand{\bidelta}{\boldsymbol{\delta}}\newcommand{\bivarepsilon}{\boldsymbol{\varepsilon}}\newcommand{\bizeta}{\boldsymbol{\zeta}}\newcommand{\bieta}{\boldsymbol{\eta}}\newcommand{\bitheta}{\boldsymbol{\theta}}\newcommand{\biiota}{\boldsymbol{\iota}}\newcommand{\bikappa}{\boldsymbol{\kappa}}\newcommand{\bilambda}{\boldsymbol{\lambda}}\newcommand{\bimu}{\boldsymbol{\mu}}\newcommand{\binu}{\boldsymbol{\nu}}\newcommand{\bixi}{\boldsymbol{\xi}}\newcommand{\biomicron}{\boldsymbol{\micron}}\newcommand{\bipi}{\boldsymbol{\pi}}\newcommand{\birho}{\boldsymbol{\rho}}\newcommand{\bisigma}{\boldsymbol{\sigma}}\newcommand{\bitau}{\boldsymbol{\tau}}\newcommand{\biupsilon}{\boldsymbol{\upsilon}}\newcommand{\biphi}{\boldsymbol{\phi}}\newcommand{\bichi}{\boldsymbol{\chi}}\newcommand{\bipsi}{\boldsymbol{\psi}}\newcommand{\biomega}{\boldsymbol{\omega}}\xi$ changes with slope (*b_1_* − *b_2_*) along the vertical meridian. *b_3_* is a parameter that allows $\newcommand{\bialpha}{\boldsymbol{\alpha}}\newcommand{\bibeta}{\boldsymbol{\beta}}\newcommand{\bigamma}{\boldsymbol{\gamma}}\newcommand{\bidelta}{\boldsymbol{\delta}}\newcommand{\bivarepsilon}{\boldsymbol{\varepsilon}}\newcommand{\bizeta}{\boldsymbol{\zeta}}\newcommand{\bieta}{\boldsymbol{\eta}}\newcommand{\bitheta}{\boldsymbol{\theta}}\newcommand{\biiota}{\boldsymbol{\iota}}\newcommand{\bikappa}{\boldsymbol{\kappa}}\newcommand{\bilambda}{\boldsymbol{\lambda}}\newcommand{\bimu}{\boldsymbol{\mu}}\newcommand{\binu}{\boldsymbol{\nu}}\newcommand{\bixi}{\boldsymbol{\xi}}\newcommand{\biomicron}{\boldsymbol{\micron}}\newcommand{\bipi}{\boldsymbol{\pi}}\newcommand{\birho}{\boldsymbol{\rho}}\newcommand{\bisigma}{\boldsymbol{\sigma}}\newcommand{\bitau}{\boldsymbol{\tau}}\newcommand{\biupsilon}{\boldsymbol{\upsilon}}\newcommand{\biphi}{\boldsymbol{\phi}}\newcommand{\bichi}{\boldsymbol{\chi}}\newcommand{\bipsi}{\boldsymbol{\psi}}\newcommand{\biomega}{\boldsymbol{\omega}}\xi$ to differ between the upper and lower visual fields. When *b_3_* is positive, $\newcommand{\bialpha}{\boldsymbol{\alpha}}\newcommand{\bibeta}{\boldsymbol{\beta}}\newcommand{\bigamma}{\boldsymbol{\gamma}}\newcommand{\bidelta}{\boldsymbol{\delta}}\newcommand{\bivarepsilon}{\boldsymbol{\varepsilon}}\newcommand{\bizeta}{\boldsymbol{\zeta}}\newcommand{\bieta}{\boldsymbol{\eta}}\newcommand{\bitheta}{\boldsymbol{\theta}}\newcommand{\biiota}{\boldsymbol{\iota}}\newcommand{\bikappa}{\boldsymbol{\kappa}}\newcommand{\bilambda}{\boldsymbol{\lambda}}\newcommand{\bimu}{\boldsymbol{\mu}}\newcommand{\binu}{\boldsymbol{\nu}}\newcommand{\bixi}{\boldsymbol{\xi}}\newcommand{\biomicron}{\boldsymbol{\micron}}\newcommand{\bipi}{\boldsymbol{\pi}}\newcommand{\birho}{\boldsymbol{\rho}}\newcommand{\bisigma}{\boldsymbol{\sigma}}\newcommand{\bitau}{\boldsymbol{\tau}}\newcommand{\biupsilon}{\boldsymbol{\upsilon}}\newcommand{\biphi}{\boldsymbol{\phi}}\newcommand{\bichi}{\boldsymbol{\chi}}\newcommand{\bipsi}{\boldsymbol{\psi}}\newcommand{\biomega}{\boldsymbol{\omega}}\xi$ is greater in the upper hemifield, and when *b_3_* is negative, $\newcommand{\bialpha}{\boldsymbol{\alpha}}\newcommand{\bibeta}{\boldsymbol{\beta}}\newcommand{\bigamma}{\boldsymbol{\gamma}}\newcommand{\bidelta}{\boldsymbol{\delta}}\newcommand{\bivarepsilon}{\boldsymbol{\varepsilon}}\newcommand{\bizeta}{\boldsymbol{\zeta}}\newcommand{\bieta}{\boldsymbol{\eta}}\newcommand{\bitheta}{\boldsymbol{\theta}}\newcommand{\biiota}{\boldsymbol{\iota}}\newcommand{\bikappa}{\boldsymbol{\kappa}}\newcommand{\bilambda}{\boldsymbol{\lambda}}\newcommand{\bimu}{\boldsymbol{\mu}}\newcommand{\binu}{\boldsymbol{\nu}}\newcommand{\bixi}{\boldsymbol{\xi}}\newcommand{\biomicron}{\boldsymbol{\micron}}\newcommand{\bipi}{\boldsymbol{\pi}}\newcommand{\birho}{\boldsymbol{\rho}}\newcommand{\bisigma}{\boldsymbol{\sigma}}\newcommand{\bitau}{\boldsymbol{\tau}}\newcommand{\biupsilon}{\boldsymbol{\upsilon}}\newcommand{\biphi}{\boldsymbol{\phi}}\newcommand{\bichi}{\boldsymbol{\chi}}\newcommand{\bipsi}{\boldsymbol{\psi}}\newcommand{\biomega}{\boldsymbol{\omega}}\xi$ is greater in the lower hemifield. Note that *b_3_* does not affect $\newcommand{\bialpha}{\boldsymbol{\alpha}}\newcommand{\bibeta}{\boldsymbol{\beta}}\newcommand{\bigamma}{\boldsymbol{\gamma}}\newcommand{\bidelta}{\boldsymbol{\delta}}\newcommand{\bivarepsilon}{\boldsymbol{\varepsilon}}\newcommand{\bizeta}{\boldsymbol{\zeta}}\newcommand{\bieta}{\boldsymbol{\eta}}\newcommand{\bitheta}{\boldsymbol{\theta}}\newcommand{\biiota}{\boldsymbol{\iota}}\newcommand{\bikappa}{\boldsymbol{\kappa}}\newcommand{\bilambda}{\boldsymbol{\lambda}}\newcommand{\bimu}{\boldsymbol{\mu}}\newcommand{\binu}{\boldsymbol{\nu}}\newcommand{\bixi}{\boldsymbol{\xi}}\newcommand{\biomicron}{\boldsymbol{\micron}}\newcommand{\bipi}{\boldsymbol{\pi}}\newcommand{\birho}{\boldsymbol{\rho}}\newcommand{\bisigma}{\boldsymbol{\sigma}}\newcommand{\bitau}{\boldsymbol{\tau}}\newcommand{\biupsilon}{\boldsymbol{\upsilon}}\newcommand{\biphi}{\boldsymbol{\phi}}\newcommand{\bichi}{\boldsymbol{\chi}}\newcommand{\bipsi}{\boldsymbol{\psi}}\newcommand{\biomega}{\boldsymbol{\omega}}\xi$ on the vertical meridian (where *φ* = ±π/2), a region of visual space we were unable to test because of the logic of our left/right 2AFC task. All parameters were fit by minimizing the summed, absolute values of differences between the log-transformed measured and predicted detection thresholds.

## Results

**Figure 3:**
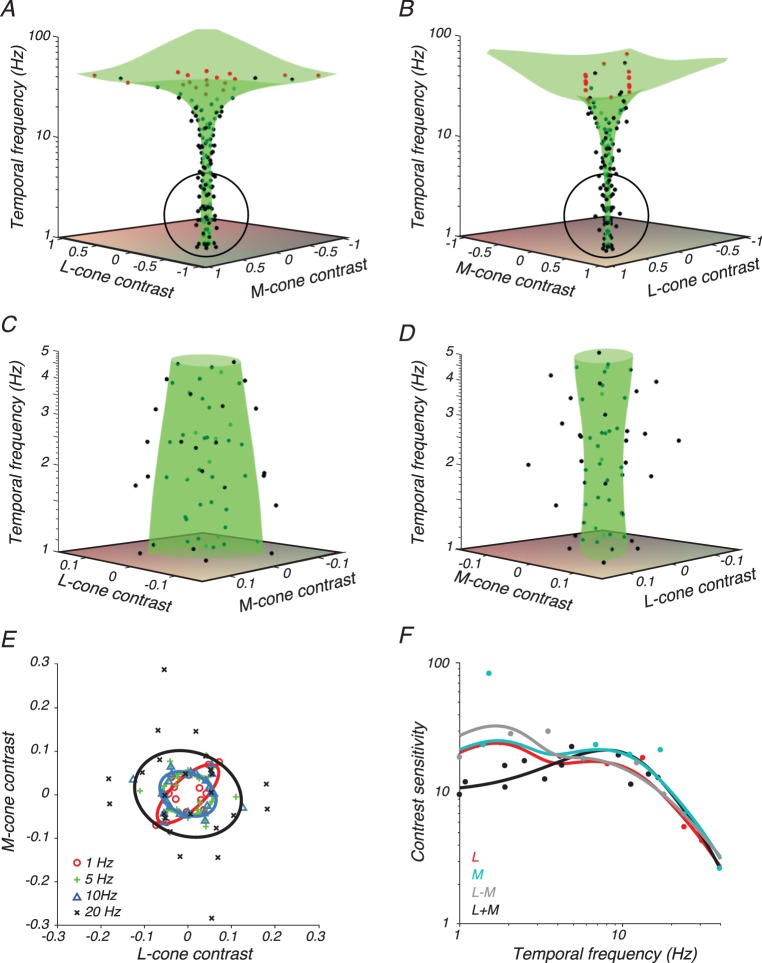
Data from subject M2 and model fit. (A–D) Contrast detection thresholds (black points) on trials in which the stimulus appeared 5° from the fixation point on the horizontal meridian. Stimulus directions for which a threshold could not be measured because of limitations of the display gamut are plotted at the gamut edge (red points). Surfaces are best fits of Equation 7 (green). (A) Stimulus space oriented so that the L+M axis is in the plane of the page. (B) Stimulus space oriented so that the L−M axis is in the plane of the page. (C, D) Magnified views of the circled portion of A and B, respectively. (E) Cross sections through the surfaces in A–D parallel to the LM plane at 1 Hz (red), 5 Hz (green), 10 Hz (blue), and 20 Hz (black). Detection thresholds (symbols) were collected from bins that spanned the nominal temporal frequency ± a factor of 1.5. (F) Contrast sensitivity measurements (points) and 1-D functions from the model fit (curves) in the L-cone direction (red), M-cone direction (cyan), L−M direction (gray), and L+M direction (black). Data points were collected from bins that spanned the nominal color direction ± 10°.

We measured contrast detection thresholds of two monkey and two human subjects as a function of three variables: temporal frequency, angle in the LM plane, and location in the visual field. Thresholds of subject M2 (Figure 3), measured at screen location *r* = 5, *φ* = 0, capture many features of this broader data set.

Thresholds generally increased with temporal frequency, as shown by the flaring of the data points and the fitted surface along the temporal frequency axis (Figure 3A, B). To show the effects of color direction, the data have been plotted twice: once rotated so that the L+M axis is in the plane of the page (Figure 3A) and once rotated so that the L−M axis is in the plane of the page (Figure 3B).

**Figure 4:**
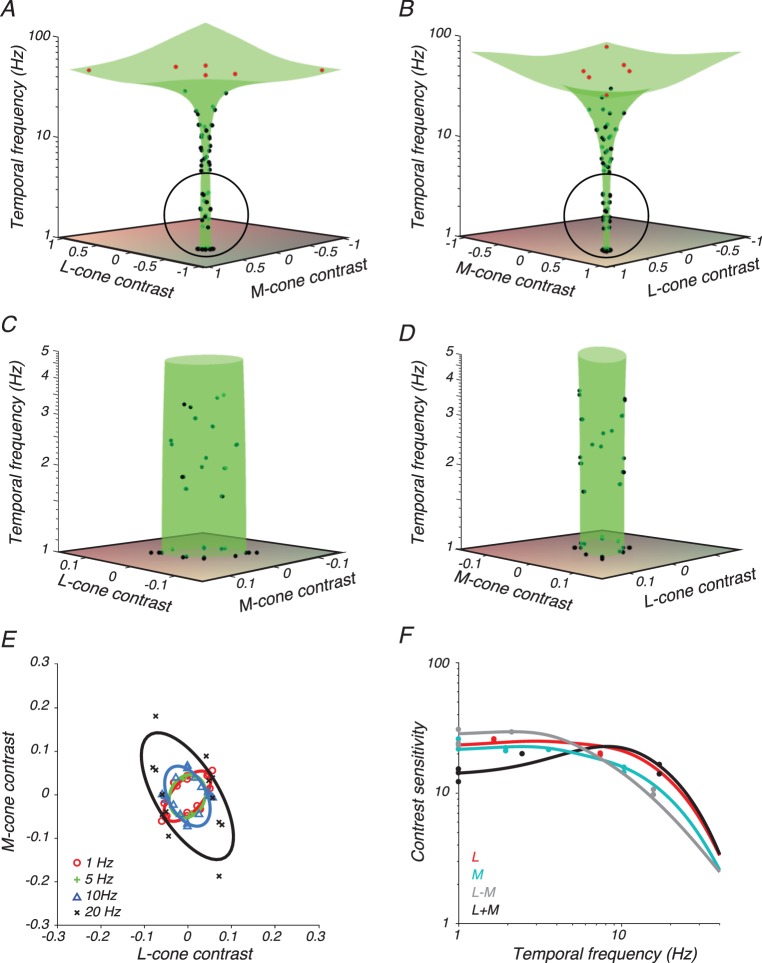
Data and model fits from subject H1 with conventions as in Figure 3.

Detection thresholds for low temporal frequency L+M modulations were greater than for low temporal frequency L−M modulations, as expected (Stromeyer et al., 1985). This feature of the data is manifest in the greater width of the fitted threshold surface in the L+M direction (Figure 3C) than in the L−M direction (Figure 3D). It can also be seen in slices through the detection threshold surface fit: detection ellipses (Figure 3E) and contrast sensitivity functions (Figure 3F). The bump in RG sensitivity at ∼2 Hz (Figure 3F) was a consequence of noisy data fit with a flexible model. It was not present in data from M2 at other locations nor in equivalent data from human subject H1 (Figure 4).

### Modeling contrast sensitivity at individual visual field locations

**Figure 5:**
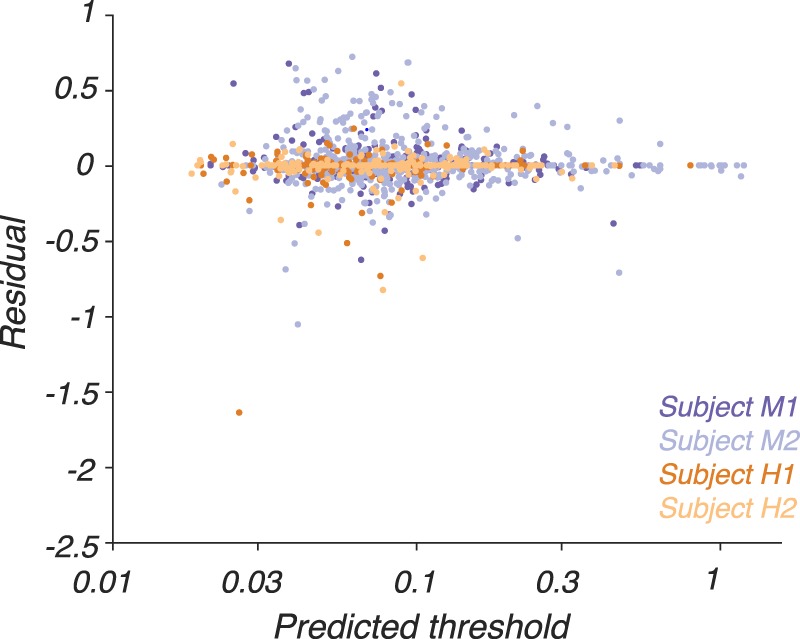
Residuals from the 13-parameter model fits (Equation 7) as a function of predicted threshold. Residuals are defined as log_10_(measured threshold) − log_10_ (predicted threshold), where both measured and predicted thresholds are in units of stimulus modulation amplitude (Equation 4). Models were fit independently to data collected at each visual field location. Residuals from each subject are plotted in a different color (see inset).

For each observer, we measured detection thresholds at 11 to 21 locations in the visual field and fit the data independently at each location. Each of these fits contains 13 parameters: six that control the contrast sensitivity of the LUM mechanism, six that control the contrast sensitivity of the RG mechanism, and one that controls the L:M ratio of the LUM mechanism (see Equation 7 in the Methods section). We iteratively refit data from each location using solutions from every other location as initial guesses to the solver (MATLAB, MathWorks, Natick, MA; fmincon) until none of the fits improved. We confirmed that the final model described the data well in the sense that the distribution of the residuals was centered on zero, was narrow, and depended little on predicted threshold (Figure 5). A subtle decrease in the variance of the residuals with predicted threshold may be due to the exclusion from this analysis of thresholds beyond the display gamut, which occur preferentially under high predicted-threshold conditions.

Describing detection thresholds at each visual field location independently had two significant shortcomings. First, the model overfit the data; many parameters were used to fit few data points. Second, predictions were made only at locations in the visual field at which thresholds had been measured. In the next section, we describe an extension of the model with fewer parameters that generalizes to a continuum of visual field locations.

### Modeling contrast sensitivity across visual field locations

To extend the model, we first looked for patterns in the fitted values of the 13 model parameters across locations in the visual field. For each subject, we plotted the best-fit value of each parameter as a function of location in the visual field and inspected the plots to identify trends. The parameters *ξ_LUM_* and *ξ_RG_*, which specify the sensitivity of the LUM and RG mechanisms, respectively, stood out as strongly eccentricity dependent (Equation 6, data not shown). These two parameters were therefore allowed to change with visual field location in all model variants described below.

We considered the possibility that allowing *n_LUM_* (Equation 5), *n_RG_* (Equation 5), or *θ* (Equation 7) to vary across the visual field, in addition to *ξ_LUM_* and *ξ_RG_*, would improve the model fit. *n_LUM_* and *n_RG_* affect the slope of the high-frequency roll-off of the LUM and RG mechanisms, respectively, and *θ* affects the L:M cone weighting to the LUM mechanism. We fit the data from each subject using models in which *ξ_LUM_* and *ξ_RG_* and, optionally, one of the set (*n_LUM_*, *n_RG_*, and *θ*), were allowed to vary across location. All other parameters were constrained to have the same value at every location. Individual threshold measurements were held out from each fit and used to calculate prediction errors from each model.

Prediction errors were similar when computed from models that allowed *n_LUM_*, *n_RG_*, or *θ* to vary as from a model that did not (one-sided Wilcoxon tests, *p* > 0.1 in all 12 cases: 3 models × 4 subjects). These results are consistent with the idea that the overall sensitivity of the LUM and RG mechanisms, but not *n_LUM_*, *n_RG_*, or *θ*, varies across the region of the visual field that we probed. We therefore focused exclusively on models for which only *ξ_LUM_* and *ξ_RG_* changed with visual field location. In the next section, we discuss the parametric form of this dependence.

### Parametric description of *ξ_LUM_* and *ξ_RG_* across visual space

**Figure 6:**
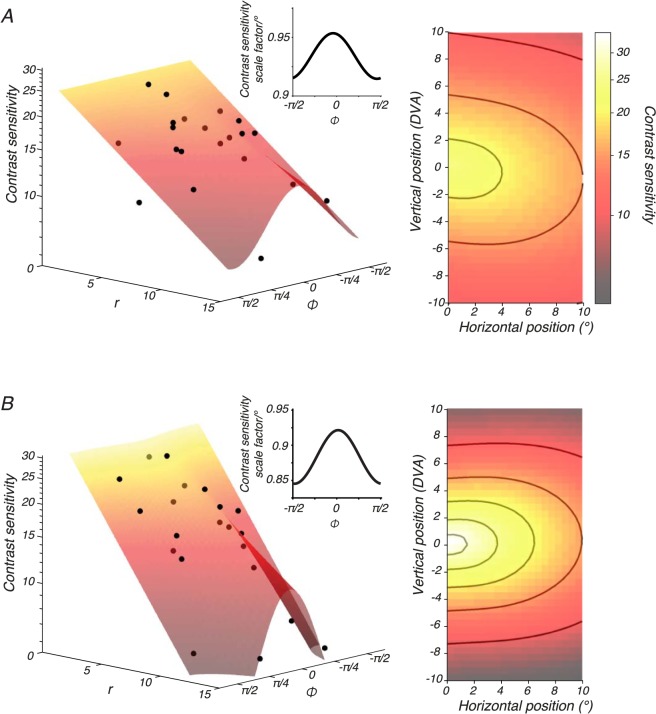
Variations in ξ_LUM_ and ξ_RG_ across the visual field. Data from subject M1 were fitted with a model in which all of the parameters except ξ_LUM_ and ξ_RG_ were fixed across visual field locations. Left: LUM (A, black dots) and RG (B, black dots) contrast sensitivity as a function of visual field location, parameterized by r and φ. Contrast sensitivity is the reciprocal of detection threshold in units of stimulus modulation amplitude (Equation 4). To facilitate comparison between LUM and RG, contrast sensitivity was evaluated at 6 Hz, which is the frequency at which the components of the fitted model apart from ξ_LUM_ and ξ_RG_ confer equal sensitivity. Surfaces were fit with Equation 10. Insets show the slope of the modelled contrast sensitivity decline as a function of φ (e.g., for each degree of eccentricity along the horizontal meridian, LUM contrast sensitivity drops by a factor of 0.95). Right: Surface fits from the left rendered as a heat map with visual field location represented in degrees of visual angle. The color bar applies to both top and bottom panels. Contours in A are 20, 15, and 10. Contours in B are 30, 25, 20, 15, and 10.

Contrast sensitivity for all subjects dropped more quickly along the vertical meridian than along the horizontal meridian for both LUM (Figure 6A) and RG (Figure 6B). We modeled this pattern in the data with Equation 10 (see the Methods section) and considered four variants of the model. Each model variant applied different constraints to *b_3_*, which controls the asymmetry of detection thresholds above and below the horizontal meridian. In the “symmetric” variant, sensitivity was forced to be symmetric in the upper and lower visual fields (*b_3_* = 0 for both *ξ_LUM_* and *ξ_RG_*). In the “yoked” variant, the upper and lower visual field asymmetry was constrained to be identical for both mechanisms (a single *b_3_* parameter was shared by *ξ_LUM_* and *ξ_RG_*). In the “luminance-only” variant, LUM sensitivity, but not RG sensitivity, was allowed to differ between upper and lower visual fields (*b_3_* = 0 for *ξ_RG_*). In the “unconstrained” variant, LUM and RG sensitivity was allowed to differ independently and asymmetrically in the upper and lower visual fields (*b_3_* was fit separately for *ξ_LUM_* and *ξ_RG_*).

**Figure 7:**
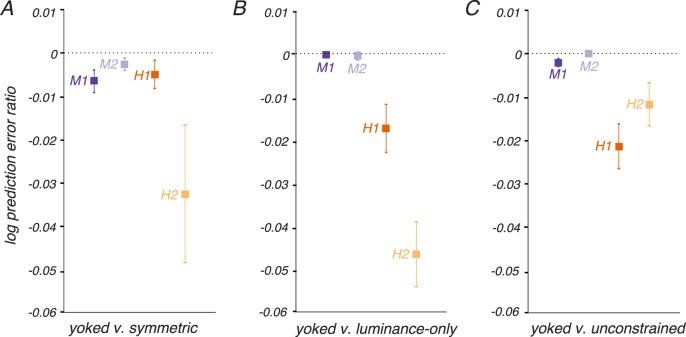
Cross-validated model comparisons. Individual threshold measurements were withheld from fitting and used to calculate prediction errors for four models: symmetric (b_3_ = 0 for both ξ_LUM_ and ξ_RG_), yoked (a single b_3_ parameter was shared by ξ_LUM_ and ξ_RG_), luminance-only (b_3_ = 0 for ξ_RG_), and unconstrained (b_3_ was fit separately for ξ_LUM_ and ξ_RG_). The prediction error is quantified as log_10_(measured threshold) − log_10_(predicted threshold), where threshold is measured in units of stimulus modulation amplitude (Equation 4). The ratio of prediction errors between models was calculated for each threshold measurement. Negative log prediction error ratios indicate that the yoked model produced lower prediction errors than the alternative model. Points and error bars indicate medians and bootstrap estimates of standard error. More data were collected from monkeys than humans, resulting in smaller error bars for monkeys.

We compared these model variants using a leave-one-out, cross-validated analysis of prediction error similar to the analysis of *n_LUM_*, *n_RG_*, and *θ* previously described. We held out individual threshold measurements, fit the four models (symmetric, yoked, luminance-only, and unconstrained) to the remaining data, recorded prediction errors between the model fits and the held-out data point, and repeated this process for each threshold measurement. The model with the lowest prediction errors, for all subjects, was the yoked variant (Figure 7).

**Figure 8:**
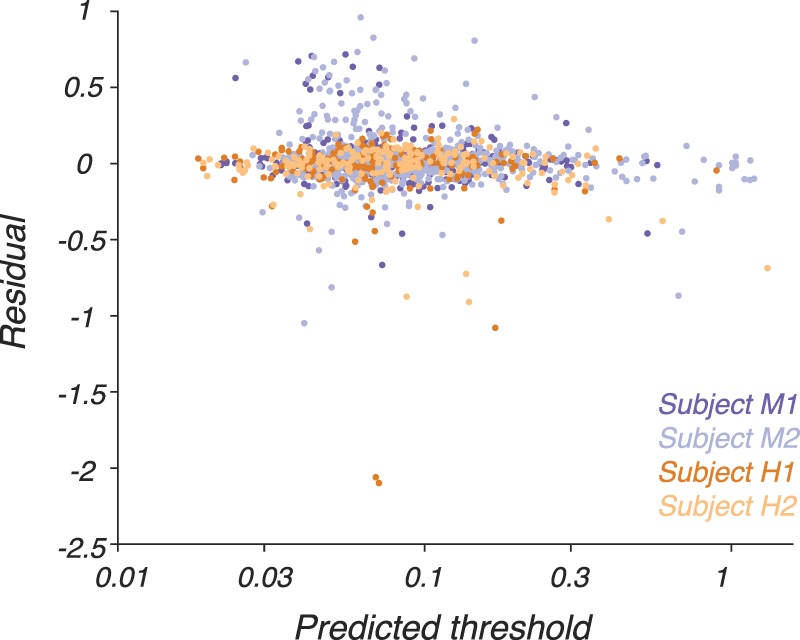
Residuals, defined as log_10_(measured threshold) − log_10_(predicted threshold), from the 18-parameter model fits (Equation 10) as a function of predicted threshold. Conventions are as in Figure 4.

The yoked model contained 18 parameters: 13 that governed sensitivity as a function of temporal frequency and color direction, and five that governed changes in two of the 13 parameters (*ξ_LUM_* and *ξ_RG_*) across the visual field. Residuals from these model fits, plotted as a function of predicted threshold, were similar to those obtained when a separate 13-parameter model was fitted to the data at each screen location individually despite the 8- to 15-fold reduction in the number of parameters (Figure 8, compare to Figure 5).

The median ratio between the measured and predicted thresholds was 1.00, indicating that the predictions were not systematically biased upward or downward. The 10th and 90th percentiles of the ratios were 0.77 and 1.40, respectively, indicating that 80% of the measured thresholds were within a factor of ∼0.7 of the predictions. We conclude that the model fit most of the data accurately.

### Analysis of residuals

If the model were specified perfectly, we would expect the residuals to be independent and identically distributed across all combinations of temporal frequency, color direction, and visual field location. Testing this hypothesis is difficult given the number of independent variables, but to confirm the absence of strong patterns in the residuals, we performed two additional analyses. In each analysis, we pooled residuals across two of the stimulus variables (e.g., *r* and *φ* location in the visual field) and examined them as a function of the remaining two (e.g., color direction and temporal frequency).

**Figure 9:**
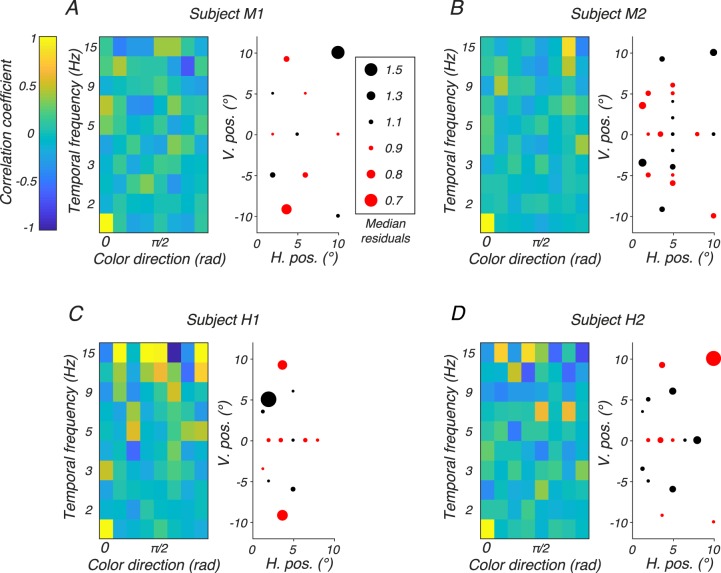
Analysis of residuals from the 18-parameter model fits. Panels A, B, C, and D show results from subjects M1, M2, H1, and H2, respectively, and each panel shows results from two analyses. Left: Autocorrelation of median residuals as a function of color direction (abscissa) and temporal frequency (ordinate). Color represents Pearson's correlation coefficient (for color bar, see inset in A). Right: Magnitude and sign of median residual (for dot size and color, see inset in A) as a function of stimulus location in the visual field. The median residual is the median of the distribution of ratios between the measured and predicted thresholds.

First, we collapsed residuals across visual field locations and calculated the autocorrelation of median residuals as a function of color direction and temporal frequency (Figure 9, left side of each panel). This autocorrelation function was fairly flat for all subjects, consistent with independent residuals across temporal frequency and color direction.

Second, we plotted median residuals as a function of location (Figure 9, right side of each panel). Residuals for subjects M1 and H1 had little discernable structure. On the other hand, the model systematically overestimated subject M2's sensitivity near the horizontal meridian along the line *h* = 5° and underestimated it further from the horizontal meridian (Figure 9B). This pattern is probably due to task training: visual field locations at which sensitivity was overestimated were tested earlier than locations at which sensitivity was underestimated. For subject H2, the assumption that contrast sensitivity decays exponentially along the horizontal meridian is imperfect. For this subject, contrast sensitivity drops more gradually over the central 5° of the horizontal meridian than predicted from exponential decay (Figure 9D; Equation 10).

### Human-monkey comparison

**Figure 10:**
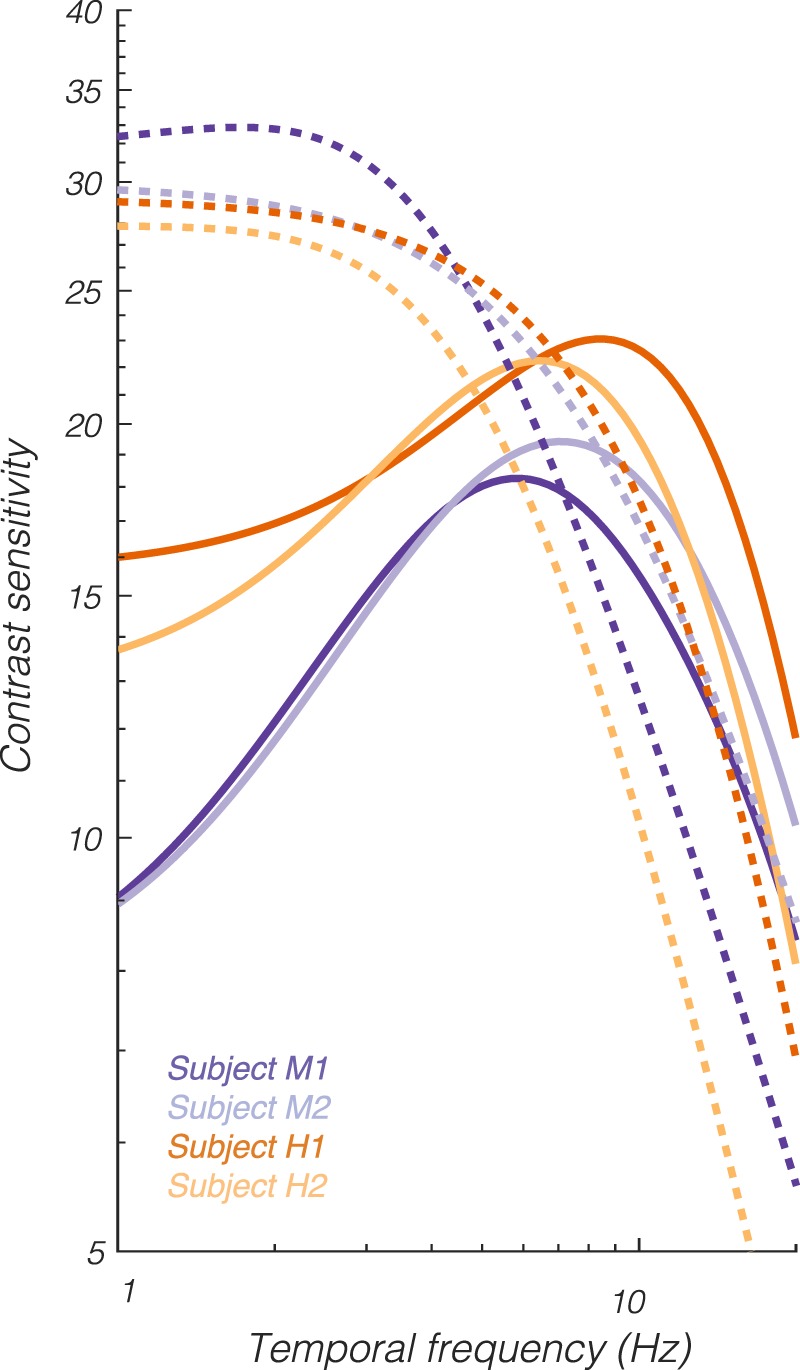
Temporal contrast sensitivity functions from the 18-parameter (yoked) model fits for each subject evaluated at screen location r = 5, φ = 0 in the L+M (solid) and L−M (dashed) directions.

As expected from previous studies, human and monkey temporal contrast sensitivity was similar (De Valois et al., 1974; Merigan, 1980). Here, we extended these results to all directions in the LM plane and a variety of locations in the visual field from 2° to 14°. To test quantitatively for differences in temporal contrast sensitivity between humans and monkeys, we took the raw contrast sensitivity measurements for each subject, normalized them within each visual field location, and then pooled them across locations. Normalized luminance contrast sensitivity was greater for humans than monkeys from 1 to 1.5 Hz (two-way analysis of variance with subject as a random effect, *p* = 0.056). Model fits for each subject, evaluated at location *r* = 5°, *φ* = 0, illustrate this difference (Figure 10).

## Discussion

We measured the contrast detection thresholds of two humans and two monkeys as a function of three variables: temporal frequency, location in the visual field, and color direction in the LM plane. We built a model that successfully described thresholds for all observers over the range of stimulus variables tested (1–60 Hz, 2°–14° of eccentricity, and all color directions within the LM plane).

We obtained three main results. First, the model fitted contrast detection threshold data from both humans and monkeys with small adjustments to the parameters (Table 2). This confirms the similarity between monkey and human luminance contrast sensitivity and extends these results across color directions in the LM plane and visual field locations. Second, the model did not require complex interactions among parameters to fit the data adequately. This is not trivial: As the number of stimulus variables increases linearly, the number of variable combinations increases exponentially. Theoretically, for example, sensitivity to L-cone modulations in the upper visual field could have been poorly predicted by a model that assumes independent contributions of color direction and screen location to contrast sensitivity, but this was not the case. Third, we found, using the model as a guide, that monkeys were only half as sensitive to low-frequency luminance modulations as humans. A retrospective look at data from a previous study confirms this result, although this difference was not previously emphasized (Merigan, 1980, their figure 3).

**Table 2:** Parameter values from final fitted models.

	Subject M1	Subject M2	Subject H1	Subject H2
$\newcommand{\bialpha}{\boldsymbol{\alpha}}\newcommand{\bibeta}{\boldsymbol{\beta}}\newcommand{\bigamma}{\boldsymbol{\gamma}}\newcommand{\bidelta}{\boldsymbol{\delta}}\newcommand{\bivarepsilon}{\boldsymbol{\varepsilon}}\newcommand{\bizeta}{\boldsymbol{\zeta}}\newcommand{\bieta}{\boldsymbol{\eta}}\newcommand{\bitheta}{\boldsymbol{\theta}}\newcommand{\biiota}{\boldsymbol{\iota}}\newcommand{\bikappa}{\boldsymbol{\kappa}}\newcommand{\bilambda}{\boldsymbol{\lambda}}\newcommand{\bimu}{\boldsymbol{\mu}}\newcommand{\binu}{\boldsymbol{\nu}}\newcommand{\bixi}{\boldsymbol{\xi}}\newcommand{\biomicron}{\boldsymbol{\micron}}\newcommand{\bipi}{\boldsymbol{\pi}}\newcommand{\birho}{\boldsymbol{\rho}}\newcommand{\bisigma}{\boldsymbol{\sigma}}\newcommand{\bitau}{\boldsymbol{\tau}}\newcommand{\biupsilon}{\boldsymbol{\upsilon}}\newcommand{\biphi}{\boldsymbol{\phi}}\newcommand{\bichi}{\boldsymbol{\chi}}\newcommand{\bipsi}{\boldsymbol{\psi}}\newcommand{\biomega}{\boldsymbol{\omega}}{\zeta _{LUM}}$	0.56	0.69	0.52	0.42
*n_LUM (filter 1)_*	4.05	2.87	3.86	4.05
*n_LUM (filter 2)_*	5.45	2.87	5.36	9.03
*T_LUM (filter 1)_*	5 × 10^−3^	7 × 10^−3^	8 × 10^−3^	6 × 10^−3^
*T_LUM (filter 2)_*	0.09	0.13	0.35	1.00
$\newcommand{\bialpha}{\boldsymbol{\alpha}}\newcommand{\bibeta}{\boldsymbol{\beta}}\newcommand{\bigamma}{\boldsymbol{\gamma}}\newcommand{\bidelta}{\boldsymbol{\delta}}\newcommand{\bivarepsilon}{\boldsymbol{\varepsilon}}\newcommand{\bizeta}{\boldsymbol{\zeta}}\newcommand{\bieta}{\boldsymbol{\eta}}\newcommand{\bitheta}{\boldsymbol{\theta}}\newcommand{\biiota}{\boldsymbol{\iota}}\newcommand{\bikappa}{\boldsymbol{\kappa}}\newcommand{\bilambda}{\boldsymbol{\lambda}}\newcommand{\bimu}{\boldsymbol{\mu}}\newcommand{\binu}{\boldsymbol{\nu}}\newcommand{\bixi}{\boldsymbol{\xi}}\newcommand{\biomicron}{\boldsymbol{\micron}}\newcommand{\bipi}{\boldsymbol{\pi}}\newcommand{\birho}{\boldsymbol{\rho}}\newcommand{\bisigma}{\boldsymbol{\sigma}}\newcommand{\bitau}{\boldsymbol{\tau}}\newcommand{\biupsilon}{\boldsymbol{\upsilon}}\newcommand{\biphi}{\boldsymbol{\phi}}\newcommand{\bichi}{\boldsymbol{\chi}}\newcommand{\bipsi}{\boldsymbol{\psi}}\newcommand{\biomega}{\boldsymbol{\omega}}{\zeta _{RG}}$	0.15	0.00	0.61	0.40
*n_RG (filter 1)_*	1.32	1.32	1.80	3.20
*n_RG (filter 2)_*	4.74	2.70	1.87	3.45
*T_RG (filter 1)_*	0.03	0.02	0.03	0.01
*T_RG (filter 2)_*	1.00	0.14	0.77	1.00
*θ*	0.78	0.80	0.49	0.71
*b_0 LUM_*	1.33	1.51	1.59	1.62
*b_1 LUM_*	−0.03	−0.03	−0.04	−0.05
*b_2 LUM_*	5 × 10^−3^	6 × 10^−3^	4 × 10^−3^	8 × 10^−3^
*b_0 RG_*	1.82	1.66	2.30	2.14
*b_1 RG_*	−0.06	−0.05	−0.10	−0.12
*b_2 RG_*	0.013	0.02	0.01	0.03
*b_3_*	−6 × 10^−4^	1 × 10^−3^	−7 × 10^−3^	−1 × 10^−3^

Subjects M1, H1, and H2 were heavily trained on the task before data collection began (M1 is monkey A and H2 is human G from Lindbloom-Brown et al., 2014). Subject M2 was the least heavily trained subject but exhibited similar contrast sensitivity to the others, suggesting that all four subjects had attained near-asymptotic performance. Longer training periods would likely have been necessary had we used stimuli containing S-cone increments (Gagin et al., 2014).

### Effects of eye size

Retinal illuminance depends on eye size and affects temporal contrast sensitivity (De Lange Dzn, 1958; Kelly, 1961; Snowden et al., 1995). Monkey eyes are smaller than human eyes, so their retinal illuminance is relatively high. We considered the possibility that this size difference could account for the difference between humans and monkeys in low-frequency luminance contrast sensitivity but found it unlikely. When retinal illuminance is greater than 10 Td, human detection thresholds to low-frequency luminance modulations are largely independent of illuminance when they are measured in Weber contrast (Kelly, 1961). The background of our display (producing ∼650 Td) was sufficiently intense that we would not expect low-frequency luminance contrast sensitivity to vary much, if at all, with the modest difference in retinal illuminance afforded by differences in eye size (Virsu & Lee, 1983; Smith, Lee, Pokorny, Martin, & Valberg, 1992).

### Effects of stimulus size

Adjusting stimulus size to compensate for the cortical magnification factor, a procedure called M-scaling, approximately equates detection thresholds across retinal eccentricities (Rovamo, Virsu, & Nasanen, 1978; Strasburger, Rentschler, & Jüttner, 2011). M-scaling is sufficient to equate temporal contrast sensitivity across eccentricity under some conditions (Virsu et al., 1982) but not others (Rovamo & Raninen, 1984; Raninen & Rovamo, 1986). We did not M-scale our stimuli primarily because M-scaling that equates luminance contrast detection thresholds does not equate chromatic contrast detection thresholds (Noorlander, Koenderink, den Ouden, & Edens, 1983; Rovamo & Iivanainen, 1991; Vakrou, Whitaker, McGraw, & McKeefry, 2005; Masuda & Uchikawa, 2009). An important future direction is to extend the model to multiple stimulus sizes.

### Assumptions of the model

In constructing the model, we relied heavily on results from previous studies. In this section, we present the assumptions of the model and direct the reader to the studies that supported these assumptions.

We chose a particular parametric form for the shape of the temporal contrast sensitivity function that is sufficiently flexible to fit a variety of data sets (Watson, 1986; Barten, 1993). We further assumed that detection contours in the LM plane are elliptical. This description, while demonstrably imperfect, is adequate under the stimulus conditions we used (Poirson, Wandell, Varner, & Brainard, 1990; Cole, Hine, & McIlhagga, 1994; Metha, Vingrys, & Badcock, 1994; Giulianini & Eskew, 1998). Detection thresholds of humans in the LM plane are roughly elliptical across temporal frequencies (Noorlander, Heuts, & Koenderink, 1981) and retinal locations (Stromeyer et al., 1992), and we found that this is also true for monkeys.

We assumed that the orientations and sizes of detection ellipses were given by an energy calculation on the outputs of two linear detection mechanisms (Stockman & Brainard, 2010). Noise masking reveals more than two detection mechanisms in the LM plane (Hansen & Gegenfurtner, 2013; Shepard, Swanson, McCarthy, & Eskew, 2016), but two mechanisms dominate under the conditions of our experiment (Giulianini & Eskew, 1998; Stromeyer, Thabet, Chaparro, & Kronauer, 1999). We assumed that cone weights to the two postulated detection mechanisms do not change with temporal frequency. This approximation is imperfect but is reasonable when the L- and M-cones are in similar adaptation states (Stromeyer, Cole, & Kronauer, 1987; Gegenfurtner & Hawken, 1995; Stromeyer, Chaparro, Tolias, & Kronauer, 1997; Stockman & Plummer, 2005a; Stockman & Plummer, 2005b; Stockman, Jägle, Pirzer, & Sharpe, 2008). Under the conditions of our experiment, L- and M-cones absorbed ∼8,900 and 7,400 photons/cone/s, respectively, and were therefore in an adaptation state similar to that produced by a moderate-intensity, 565-nm background. Under these conditions, flicker perception is dominated by a fast, cone-nonopponent pathway with little influence of the slow, cone-opponent pathway that might manifest as frequency-dependent cone weights to the LUM mechanism in our experiment (Stockman, Henning, Anwar, Starba, & Rider, 2018).

We also assumed that cone weights to each mechanism do not vary with retinal eccentricity. This assumption is supported by the near-constant L:M cone ratio to the RG mechanism across the visual field (Newton & Eskew, 2003; Sakurai & Mullen, 2006; Hansen, Pracejus, & Gegenfurtner, 2009) and to the LUM mechanism over the region of visual space we probed (Anderson et al., 1991; Knau, 2000). Further support for this assumption comes from our observation that allowing *θ*, the L:M ratio of the LUM mechanism in the model, to vary across the visual field did not improve prediction accuracy.

We assumed that log-transformed contrast sensitivity declines linearly with eccentricity with a slope that depends on the angle in the plane of the display screen (Robson & Graham, 1981). Our results confirmed the observation that the slope of this relationship is steeper near the vertical meridian than near the horizontal meridian (Pointer & Hess, 1989; Pointer & Hess, 1990; Abrams, Nizam, & Carrasco, 2012). Our results also confirm that low-frequency chromatic sensitivity is greater than low-frequency luminance sensitivity at the fovea (Chaparro, Stromeyer, Huang, Kronauer, & Eskew, 1993), and this relationship can reverse in the periphery due to the steeper decline in chromatic sensitivity with retinal eccentricity (Mullen, 1991; Mullen & Kingdom, 1996; Mullen & Kingdom, 2002; Mullen, Sakurai, & Chu, 2005). We found that chromatic and luminance contrast sensitivity was similarly asymmetric between upper and lower visual fields.

We assumed that the shape of the temporal contrast sensitivity function of the luminance and chromatic detection mechanisms does not change with eccentricity over the region of visual space that we probed. The assumption, which is supported by previous results (Wright & Johnston, 1983; Snowden & Hess, 1992), was built into the model by allowing only *ξ_LUM_* and *ξ_RG_* change across the visual field. We tested this assumption by asking whether allowing *n_LUM_* or *n_RG_* to vary across the visual field improved threshold predictions, and we found that it did not.

### Future directions

The contrast detection literature is vast, and extracting core principles from it and synthesizing them into a concise, accessible format is useful. For example, using the model, we can communicate large data sets with few numbers and interpolate contrast sensitivity for conditions that we did not test. The model can be used to identify stimuli for which detection is maximally or minimally constrained by signals in the early visual system (Geisler, 1989; Angueyra & Rieke, 2013; Brainard et al., 2015; Hass, Angueyra, Lindbloom-Brown, Rieke, & Horwitz, 2015) and to identify stimuli that are differentially visible between subjects. Our model spans only a few stimulus dimensions but could in principle be merged with models that predict contrast sensitivity on the basis of stimulus parameters that we did not vary (e.g., background illumination, spatial frequency, stimulus size, and S-cone modulation). Our code and data are available on GitHub (http://www.github/horwitzlab).

Our model helps to bridge the gap between neurophysiological and psychophysical studies of temporal contrast sensitivity. Measurements of neuronal responses at psychophysical detection threshold are difficult to obtain in part because detection thresholds depend on stimulus parameters in complex ways. A classic approach to this problem is to identify a suprathreshold stimulus that excites an isolated neuron strongly and then titrate a stimulus parameter (e.g., contrast) to measure psychophysical and neuronal detection thresholds simultaneously. This approach can be inefficient; psychophysical trials are longer than fixation trials, and estimating a distribution of noisy neuronal responses requires many repeated trials. Moreover, the assumption that suprathreshold stimulus preferences are predictive of neuronal sensitivity at the behavioral detection threshold may be inaccurate.

The model we present helps meet these challenges. Using the model, a battery of stimuli can be synthesized that are matched for detectability but differ in other respects (e.g., temporal frequency and color). These stimuli can be presented at the receptive fields of recorded neurons during detection task performance or passive fixation. This approach may be useful for revealing the neuronal basis of contrast sensitivity. For example, magnocellular, parvocellular, and koniocellular neurons in the lateral geniculate nucleus all respond to L+M modulations, and what contributions each makes to contrast sensitivity is poorly understood. Stimulating neurons of each type with threshold-contrast L+M modulations and comparing their relative sensitivity will provide an upper bound on each population's contribution.
